# Harnessing CD8^+^ T cell dynamics in hepatitis B virus‐associated liver diseases: Insights, therapies and future directions

**DOI:** 10.1002/ctm2.1731

**Published:** 2024-06-27

**Authors:** Bing Yue, Yuxia Gao, Yi Hu, Meixiao Zhan, Yangzhe Wu, Ligong Lu

**Affiliations:** ^1^ Guangdong Provincial Key Laboratory of Tumour Interventional Diagnosis and Treatment Zhuhai Institute of Translational Medicine Zhuhai Clinical Medical College of Jinan University (Zhuhai People's Hospital), Jinan University Zhuhai Guangdong China; ^2^ Microbiology and Immunology Department School of Medicine Faculty of Medical Science Jinan University Guangzhou Guangdong China

**Keywords:** CD8+ T Cell Dynamics, HBV‐Associated Liver Diseases, Review

## Abstract

Hepatitis B virus (HBV) infection playsa significant role in the etiology and progression of liver‐relatedpathologies, encompassing chronic hepatitis, fibrosis, cirrhosis, and eventual hepatocellularcarcinoma (HCC). Notably, HBV infection stands as the primary etiologicalfactor driving the development of HCC. Given the significant contribution ofHBV infection to liver diseases, a comprehensive understanding of immunedynamics in the liver microenvironment, spanning chronic HBV infection,fibrosis, cirrhosis, and HCC, is essential. In this review, we focused on thefunctional alterations of CD8^+^ T cells within the pathogenic livermicroenvironment from HBV infection to HCC. We thoroughly reviewed the roles ofhypoxia, acidic pH, metabolic reprogramming, amino acid deficiency, inhibitory checkpointmolecules, immunosuppressive cytokines, and the gut‐liver communication in shapingthe dysfunction of CD8^+^ T cells in the liver microenvironment. Thesefactors significantly impact the clinical prognosis. Furthermore, we comprehensivelyreviewed CD8^+^ T cell‐based therapy strategies for liver diseases,encompassing HBV infection, fibrosis, cirrhosis, and HCC. Strategies includeimmune checkpoint blockades, metabolic T‐cell targeting therapy, therapeuticT‐cell vaccination, and adoptive transfer of genetically engineered CD8^+^ T cells, along with the combined usage of programmed cell death protein‐1/programmeddeath ligand‐1 (PD‐1/PD‐L1) inhibitors with mitochondria‐targeted antioxidants.Given that targeting CD8^+^ T cells at various stages of hepatitis Bvirus‐induced hepatocellular carcinoma (HBV + HCC) shows promise, we reviewedthe ongoing need for research to elucidate the complex interplay between CD8^+^ T cells and the liver microenvironment in the progression of HBV infection toHCC. We also discussed personalized treatment regimens, combining therapeuticstrategies and harnessing gut microbiota modulation, which holds potential forenhanced clinical benefits. In conclusion, this review delves into the immunedynamics of CD8^+^ T cells, microenvironment changes, and therapeuticstrategies within the liver during chronic HBV infection, HCC progression, andrelated liver diseases.

## Graphical abstract

- HBV infection drives liver pathology from chronic hepatitis, fibrosis and cirrhosis to HCC.
- Pathological liver microenvironment induces CD8^+^ T cell dysfunction.
- Comprehensive review on CD8+ T cell–liver microenvironment interaction benefits the development of CD8+ T cell‐based immunotherapeutic strategies for liver diseases.

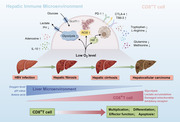

## 1 INTRODUCTION

Hepatitis B virus (HBV) infection represents a substantial global health concern, exerting profound impacts on liver function and affecting millions of individuals worldwide. The consequences of HBV infection span a spectrum of liver diseases, encompassing both acute and chronic hepatitis, progressing through stages of liver fibrosis and cirrhosis, ultimately leading to the onset of liver cancer. Notably, primary liver cancer ranks prominently among the most prevalent malignancies globally and constitutes a significant contributor to global cancer‐related mortality rates. Liver cancer incidence and mortality rates are consistently higher in males compared with females in most regions.^1^ Projections indicate a potential surge of over 55% in new liver cancer cases and deaths by the year 2040.^2^ As a prominent contributor to cancer‐related fatalities, liver cancer represents a significant public health concern. Primary liver cancer manifests in two primary forms: intrahepatic cholangiocarcinoma and hepatocellular carcinoma (HCC).^3^ HCC further subdivides into viral and non‐viral types based on aetiology, with non‐viral HCC commonly associated with alcohol abuse, obesity and smoking, while HBV‐induced HCC (HBV^+^HCC) exemplifies the viral subtype.^4^ Notably, HBV infection emerges as a major risk factor for HCC development, contributing to up to 60% of HCC cases in Asia and Africa and approximately 20% in Western countries.^3^

**FIGURE 1:**
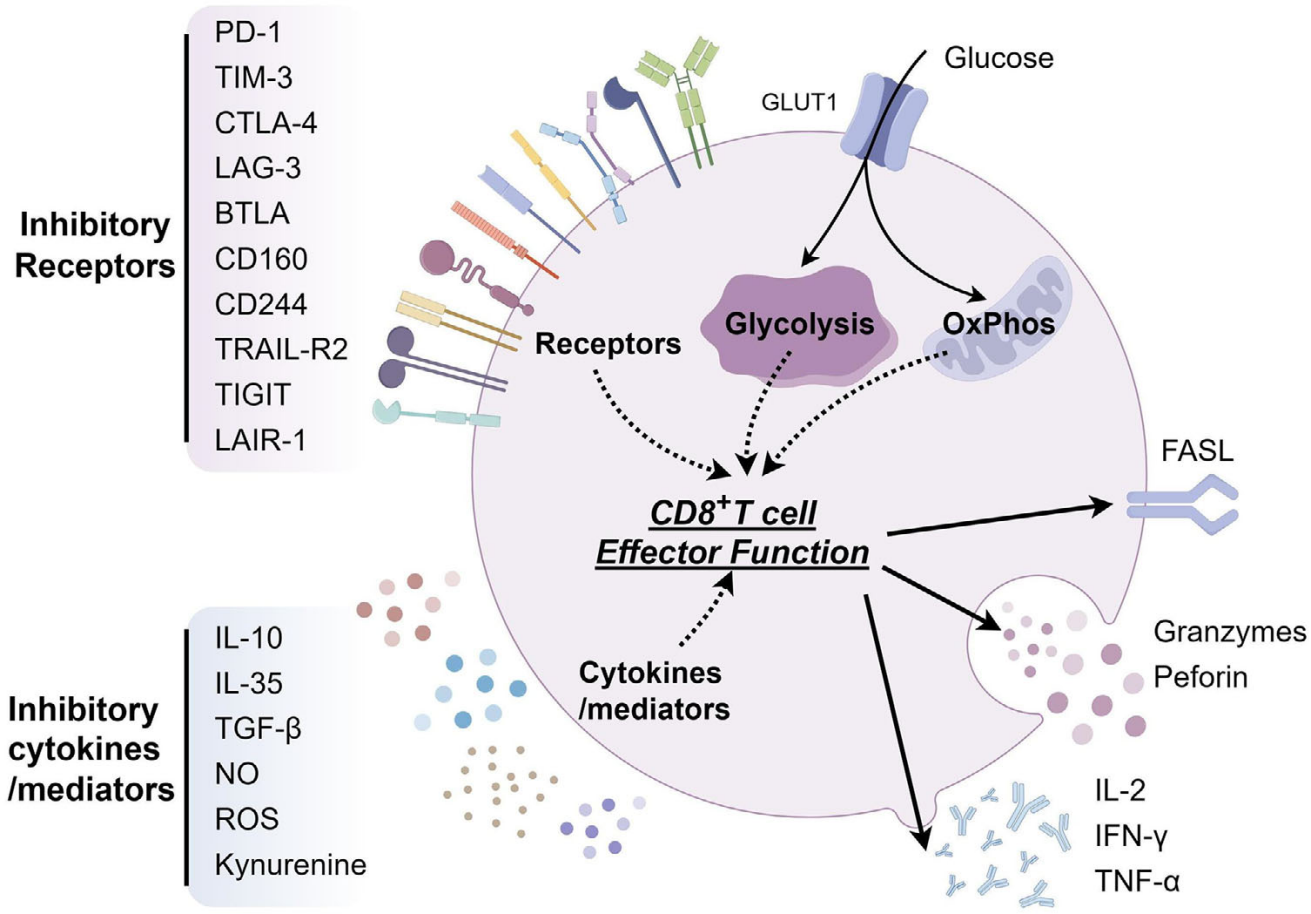
A brief overview elucidates the mechanisms underlying the induction of CD8^+^ T cell exhaustion within the pathological liver microenvironment. In response to inhibitory cytokines and mediators, CD8^+^ T cells undergo metabolic alterations and activate inhibitory receptors through intricate pathways, thereby suppressing effector functions. This suppression is distinctly characterised by diminished cytokine secretion, reduced production of cytotoxic proteins and decreased expression of FASL, and others. Nevertheless, the comprehensive elucidation of the underlying molecular mechanisms of CD8^+^ T cell dysfunction within this context is yet to be achieved. The solid arrow connected to ‘CD8^+^ T cell Effector Function’ means some of the results of the dysfunction of CD8^+^ T cell effector function, and the dashed arrow means some of the factors that lead to the dysfunction of CD8^+^ T cell effector function. PD‐1, programmed death ligand‐1; TIM‐3, T‐cell immunoglobulin mucin domain‐3; CTLA‐4, cytotoxic T‐lymphocyte‐associated protein‐4; LAG‐3, lymphocyte‐activation gene‐3; BTLA, B and T‐lymphocyte attenuator; TRAIL‐R2, TNF‐related apoptosis‐inducing ligand‐R2; LAIR‐1, leukocyte‐associated immunoglobulin‐like receptor‐1; IL‐10, interleukin‐10; IL‐35, interleukin‐35; TGF‐β, transforming growth factor‐β; NO, nitric oxide; ROS, reactive oxygen species; GLUT1, glucose transporter type 1; OXPHOS, oxidative phosphorylation; FASL, FAS ligand; IL‐2, Interleukin‐2; IFN‐γ, interferon‐γ; TNF‐α, tumour necrosis factor‐α.

Despite advancements in HBV vaccination programs, HBV infection remains a primary global risk factor for HCC. Prolonged HBV infection culminates in chronic hepatitis B (CHB), progressing to liver fibrosis, cirrhosis, and ultimately, HCC. Effective management of chronic HBV infection at any stage of progression holds potential for reducing HBV^+^HCC incidence. Extensive studies underscore the pivotal role of immune system dysregulation in HBV^+^HCC development. CD8^+^ T cells, the primary immune effectors, exert substantial influence on HCC progression by participating in the clearance of infected and malignant cells. It is acknowledged that the dysfunction of CD8^+^ T cells occurs during the progression from HBV infection to the development of HCC, and the role of CD8^+^ T cells in virus control and anti‐tumour responses is well documented as well. While the full clarification of the mechanisms driving CD8^+^ T cell exhaustion within the pathological liver microenvironment is progressively unfolding, comprehensively understanding the changes of CD8^+^ T cells in the liver microenvironment during chronic HBV infection and devising responsive strategies are imperative (Figure 1).

This review primarily explores the dynamics of the liver microenvironment throughout HBV^+^HCC progression, emphasising its impact on CD8^+^ T cell function and phenotype. The discussion spans CHB, liver fibrosis, cirrhosis and HCC, elucidating the mechanisms by which HBV infection promotes HCC development. Meanwhile, this review summarises therapeutic strategies targeting CD8^+^ T cells at different stages of HBV^+^HCC progression. Critically, the shortcomings in current HBV^+^HCC immune microenvironment and immunotherapy research are reviewed, and future prospects for this field are proposed as well. This comprehensive review aims to enhance our comprehension of the CD8^+^ T cell immunity characteristics of HBV^+^HCC, explores its underlying immune mechanisms and immunotherapeutic strategies and offers valuable insights and directions for the treatment of HCC patients.

## 2 CD8^+^ T CELL IN HBV INFECTION AND CHB

### 2.1 Immunological signatures and CD8^+^ T cell dynamics in HBV infection

The human liver serves as a pivotal hub for both systemic and local innate immunity and functions as a critical site for immune regulation.^5^ It harbours extensive populations of resident immune cells, rendering it an immunologically intricate organ essential for metabolic activities.^6^ The HBV, characterised by double‐stranded DNA, exhibits a specific liver tropism, causing damage to hepatocytes indirectly through the immune system rather than direct assault.^7^ Following entry into the liver microenvironment, HBV demonstrates the capability to infiltrate and infect liver cells, swiftly undergoing replication and dissemination throughout the hepatic tissue.^8^ The innate and adaptive immune system of the host plays a key role in the resistance to HBV infection. However, the virus has evolved characteristics that allow it to evade host immune surveillance, resulting in persistent infection and the development of CHB.^9^ Consequently, the failure to effectively activate adaptive immunity and the depletion of virus‐specific immune cells emerge as significant factors in HBV chronic infection.^7^

**FIGURE 2:**
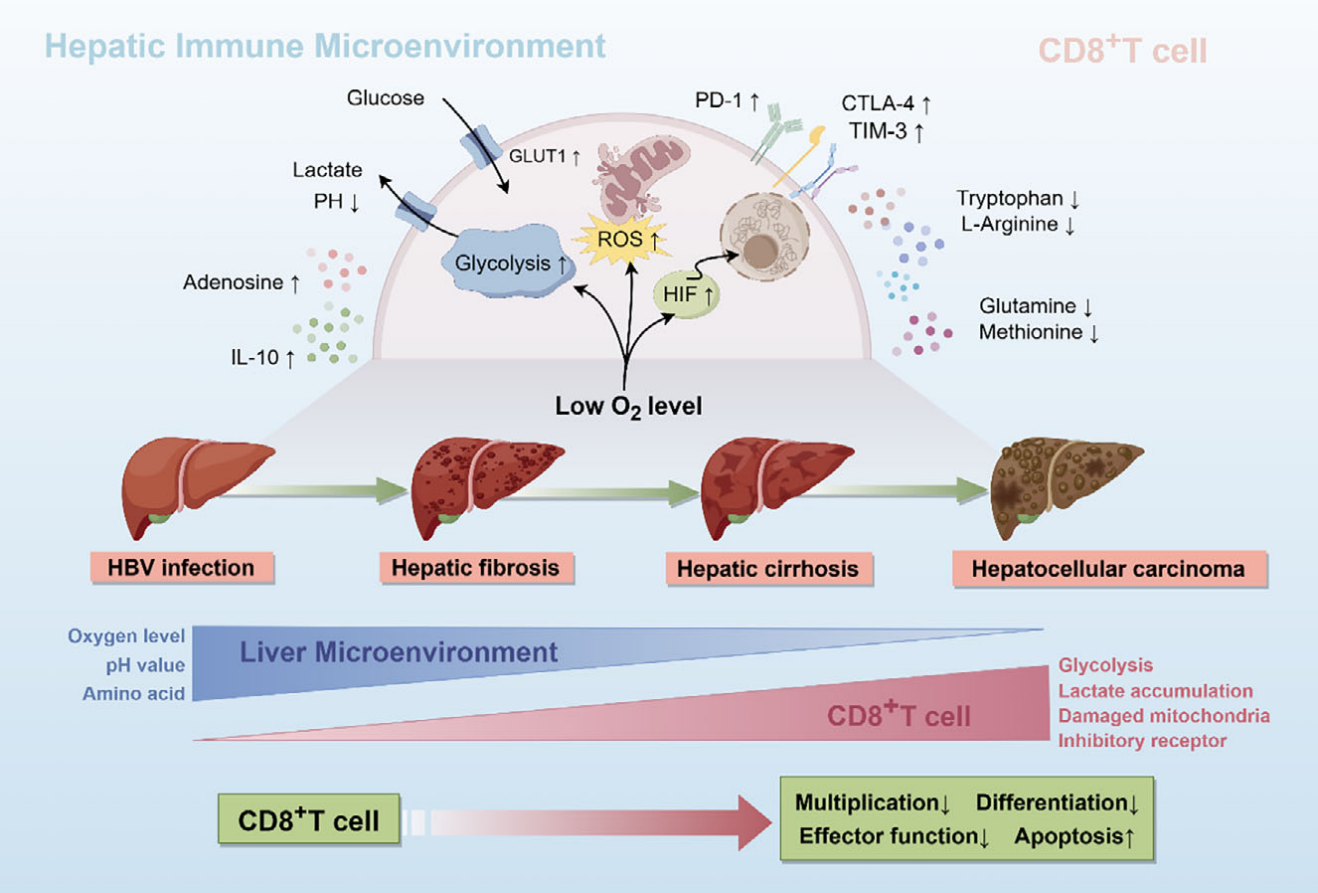
Typical alterations of CD8^+^ T cells within the liver microenvironment during progression from HBV infection to HCC. Throughout the transition from HBV infection to liver fibrosis, cirrhosis and eventual development of HCC, continuous and discernible changes occur in the liver microenvironment, marked by incremental increases in hypoxia, acidity (reduction in pH value) and amino acid deficiency. In response to these alterations in the liver microenvironment, CD8^+^ T cells undergo a series of changes as well, encompassing metabolic reprogramming, mitochondrial dysfunction, heightened expression of immunosuppressive receptors and eventual depletion of CD8^+^ T cells. IL‐10, interleukin‐10; PD‐1, programmed cell death protein‐1; CTLA‐4, cytotoxic T‐lymphocyte‐associated protein‐4; TIM‐3, T‐cell immunoglobulin mucin domain‐3; GLUT1, glucose transporter type 1; ROS, reactive oxygen species; HIF, hypoxia‐inducible factor.

CD8^+^ T cells represent crucial virus‐specific immune effectors that play a pivotal role in both HBV infection and cellular immunity. Extensive evidence indicates that during acute HBV infection, CD8^+^ T cells serve as primary effector cells responsible for viral clearance and disease pathogenesis.^10^ However, in instances where HBV infection persists and liver inflammation transitions to a chronic state, the population of CD8^+^ T cells within the liver microenvironment, critical for partial viral control and disease progression, undergoes a reduction in number and severe impairment in function. This dysfunction encompasses antigen‐specific CD8^+^ T cell‐lysing functions, primarily mediated by perforin and Granzyme B, along with non‐cytolytic functions involving the secretion of interferon (IFN)‐γ and tumour necrosis factor (TNF)‐α, capable of eliminating intracellular viruses without causing the demise of infected liver cells.^11^, ^12^ Figure 2 depicts the significant transformations in the liver microenvironment and the infiltrated CD8^+^ T cells across the spectrum from HBV infection, through fibrosis and cirrhosis, to HCC.

### 2.2 Hypoxia in HBV^+^ liver microenvironment dampens CD8^+^ T cell functions

Hypoxia plays a crucial role in the liver microenvironment during HBV infection, orchestrated by HBV‐induced stabilisation of hypoxia‐inducible factor (HIF)−1α and enhanced transactivation through the interaction of the HBV‐encoded oncogene X protein (HBx) protein with the cyclic adenosine monophosphate response element‐binding protein.^13^, ^14^ Furthermore, HBV elevates HIF‐2α expression by engaging its encoded protein, HBx, in binding with von Hippel‐Lindau protein (pVHL), thereby activating the nuclear factor kappa‐B (NF‐κB) signalling pathway.^15^

HIF, as the master regulator of the anoxic response, operates as a heterodimeric transcription factor composed of either HIF‐1α or HIF‐2α and HIF‐1β subunits.^16^ In normoxic conditions, HIF‐α interacts with the pVHL, triggering ubiquitin ligase activity and subsequent the degradation of HIF‐α.^16^ Conversely, in hypoxia, HIF‐1α engages with HIF‐1β, facilitating DNA binding at hypoxia response elements (HREs). In HBV infection, HBx binds to the nHLH/PAS domain of HIF‐1α, impeding the binding capacity of pVHL and preventing the degradation of HIF‐1α protein.^17^ Recent studies affirm the regulatory roles of both HIF‐1α and HIF‐2α in HBV transcription and indicate that HIFs can bind HBV DNA and augment HBV transcription and granulation. This suggests HBV's evolutionary adaptation to exploit the HIF‐signalling pathway for persistence in the liver's low oxygen environment.^18^, ^19^

Moreover, minor oxygen fluctuations within the liver induce HIF‐mediated hypoxic responses, affecting metabolic supply‐and‐demand ratios. Hypoxia, a significant stressor in various pathological states,^20^ including HBV infection, can hinder oxygen transport due to increased interstitial oedema and heightened inflammation‐induced oxygen consumption.^21^ Investigating the impact of oxygen tension changes on lymphocyte function reveals that under hypoxic conditions, CD8^+^ T cells maintain cytolytic activity but experience reduced proliferation, differentiation and cytokine secretion (e.g. IFN‐γ, TNF‐α and IL‐2). Although hypoxia does not compromise cytotoxic capacity, it induces IL‐10 secretion, significantly diminishing cell expansion. In summary, hypoxia alters CD8^+^ T cell activity, proliferation and cytokine secretion, accompanied by concomitant metabolic changes.

### 2.3 Metabolic reprogramming tunes antiviral responses of CD8^+^ T cells

Previous studies delve into the intricate metabolic changes and mitochondrial dysfunction observed in CD8^+^ T cells, specifically focusing on their responses during HBV infection and the subsequent impact on antiviral efficacy. Naive CD8^+^ T cells maintain metabolic stasis, relying on mitochondrial oxidative phosphorylation (OXPHOS) for energy production. Upon antigen receptor stimulation, effector CD8^+^ T cells undergo increased glycolysis, shifting from OXPHOS to glycolysis. Conversely, memory CD8^+^ T cells exhibit a preference for fatty acid oxidation (FAO) and mitochondrial metabolism.^21^ After HBV infection, CD8^+^ T cells experience metabolic alterations to meet heightened energy demands for antiviral responses. PD‐1^high^ T cells respond to HBV by up‐regulating the glucose transporter type 1 (GLUT1), a process induced by hypoxia in the liver microenvironment.^22^ Hypoxia, in turn, enhances glucose uptake by elevating the expression of glucose transporters GLUT1 and glucose transporter type 3 (GLUT3) on CD8^+^ T cell surfaces.^21^, ^23^, ^24^ HIFs play a central role in these metabolic changes, up‐regulating glycolytic enzymes and inhibiting mitochondrial oxygen consumption. This includes the activation of key metabolic targets such as lactate dehydrogenase (LDH) A, pyruvate dehydrogenase kinase 1 (PDK1) and hexokinase (HK).^24^ PDK1 inhibits mitochondrial enzyme pyruvate dehydrogenase (PDH), directing pyruvate to glycolysis and HIF‐1α expression positively correlates with glycolysis maintenance during CD8^+^ T cell responses.^25^, ^26^

Unlike more functional cytomegalovirus‐specific T cells, HBV‐specific CD8^+^ T cells, marked by elevated GLUT1 expression, are glucose dependent and unable to switch to OXPHOS. This results in mitochondrial dysfunction characterised by increased mitochondrial size and decreased potential.^22^ Impaired oxidative metabolism during hypoxia promotes the conversion of pyruvate to lactic acid, contributing to an acidic microenvironment. Overall, the reprogrammed metabolism dampens the protective responses of CD8^+^ T cells in HBV infection. Additionally, HIF activity inhibits FAO and promotes fatty acid synthesis (FAS), adapting cell metabolism to limited oxygen conditions.^27^ While PDK1‐induced acetyl‐coenzyme A (CoA) reduction seems to inhibit FAS, increased HIF activity promotes acetyl‐CoA production through glutamine, supporting adipogenesis. HIF‐driven glutathione production enhances cellular resilience to oxidative stress.^28^

The HIF‐induced metabolic changes, including enhanced glycolysis, reduced OXPHOS and mitochondrial dysfunction, significantly contribute to the compromised antiviral capabilities of CD8^+^ T cells during HBV infection. Lacking energy supply, HBV‐specific CD8^+^ T cells exhibit extensive mitochondrial aberrations, marked by elevated reactive oxygen species (ROS) and inflammatory responses, which in turn lead to cellular dysfunction.^29^, ^30^, ^31^ Consequently, the diminished energetic status impairs the migration and infiltration of CD8^+^ T cells into the liver microenvironment. Additionally, mitochondrial dysfunction such as excessive ROS and protein accumulation may inhibit proteasomal and autophagic activities,^32^ thereby impairing the effector function of the limited CD8^+^ T cell pool within the hepatic milieu.^33^, ^34^, ^35^ Collectively, existing reports have largely revealed the complex interplay between metabolic reprogramming, mitochondrial dysfunction and antiviral efficacy of CD8^+^ T cells during HBV infection. These metabolic changes directly impact the activation, infiltration and immune responses within the liver microenvironment.

### 2.4 Immune checkpoints regulate CD8^+^ T cell exhaustion

Exhausted CD8^+^ T cells in the liver expand in both chronic and acute HBV‐infected patients.^36^ Multiple factors contribute to HBV‐specific CD8^+^ T cell dysfunction, including a persistently high viral load, suppressive cytokines (IL‐10, TGF‐β), dendritic cells (DCs) and regulatory T cells (Tregs), resulting in a progressive loss of T cell function.^37^, ^38^ Key markers of HBV‐specific CD8^+^ T cell depletion include overexpression of checkpoint inhibitors, notably PD‐1, cytotoxic T‐lymphocyte‐associated protein‐4 (CTLA‐4), lymphocyte‐activation gene‐3 (LAG‐3) and T‐cell immunoglobulin mucin domain‐3 (TIM‐3). These co‐inhibitory molecules collectively contribute to CD8^+^ T cell exhaustion in patients with CHB.

While HBV‐specific CD8^+^ T cells express various inhibitory receptors, PD‐1 remains predominant. Hypoxia directly influences PD‐L1 expression through HIF‐1α, a major regulator of PD‐L1 mRNA. Hypoxia significantly up‐regulates PD‐L1 in macrophages, DCs and tumour cells, with the up‐regulation depending on HIF‐1α, not HIF‐2α. HIF‐1α directly binds to the HREs in the PD‐L1 promoter, establishing PD‐L1 as a novel direct target of HIF‐1α. Blocking PD‐L1 under hypoxia enhances myeloid‐derived suppressor cells (MDSCs)‐mediated T cell activation.^39^ Intermittent hypoxia and HIF‐1α transfection induced PD‐1/PD‐L1 crosstalk, inhibiting T cell proliferation and auto‐T lymphocyte activation, impacting the cytotoxic activity of CD8^+^ T cells.^40^ The PD‐1 pathway restricts glucose uptake, inhibits peroxisome proliferator‐activated receptor‐γ coactivator‐1α, suppresses glycolysis and OXPHOS and damages mitochondrial quality.^41^ PD‐1 clusters with T cell receptors (TCRs) upon binding to PD‐L1, forming negative co‐stimulatory micro‐clusters, inducing dephosphorylation of proximal TCR signalling molecules, suppressing T cell activation and blocking the TCR‐induced stop signal.^42^, ^43^ Antigen‐stimulated CD8^+^ T cells under these conditions exhibit substantial spare respiratory capacity, enabling prolonged survival through fatty acid metabolism.^44^ PD‐1 promotes FAO by increasing the rate‐limiting enzyme carnitine palmitoyl transferase 1A (CPT1A) and inducing lipolysis, preventing glutamine uptake and inhibiting glutaminolysis. This metabolic reprogramming leads to a memory‐like phenotype. Conversely, CTLA‐4 inhibits the expression of glutamine transporters sodium‐coupled neutral amino acid transporter (SNAT) 1 and SNAT2, glucose transporter GLUT1 and glycolysis without augmenting CPT1A and FAO.^44^ PD‐L1, the ligand of PD‐1, also inhibits HBV‐specific CD8^+^ T cell response. Maternal HBV infection induces hepatitis Be antigen (HBeAg) to up‐regulate PD‐L1 in Kupffer cells (KCs) in offspring, inhibiting HBV‐specific CD8^+^ T cell response and supporting HBV persistence after birth.^45^ This multi‐faceted overview elucidates the intricate interplay between immune exhaustion, hypoxia and metabolic shifts in HBV‐specific CD8^+^ T cells.

### 2.5 Cell–cell communication mediates apoptosis of CD8^+^ T cells

HBV‐specific CD8^+^ T cells exhibit an increased susceptibility to apoptosis. Studies have verified the elevation of pro‐apoptotic mediator B‐cell lymphoma‐2 (BCL‐2) interacting mediator of cell death (Bim) in the CD8^+^ T cells specific to HBV in patients with chronic infection, promoting the interaction with the apoptosis‐activating protein BCL‐2. The Bim‐mediated apoptosis of HBV‐specific CD8^+^ T cells results in the inability of these cell populations to persist and control viral replication. In vitro inhibition of Bim‐mediated apoptosis enhances the recovery of HBV‐specific CD8^+^ T cells.^46^ Furthermore, the apoptosis of HBV‐specific CD8^+^ T cells can be sustained through FAS and its ligand FASL interactions mediated by KCs.^47^ Signalling via TNF‐related apoptosis‐inducing ligand (TRAIL) and natural killer group 2 member D (NKG2D) receptors induced by natural killer (NK) cells also contributes to HBV‐specific CD8^+^ T cell apoptosis.^48^ Stimulation of KCs by endogenous intestinal bacterial lipopolysaccharide (LPS) leads to the generation of upstream ROS, acting as potential mediators of FASL expression in KCs. This subsequently increases FASL expression and induces apoptosis in FAS‐positive cells, such as CD8^+^ T cells. A recent study showed that activated CD8^+^ T cells are removed from the systemic circulation by the liver during liver metastasis, and activated antigen‐specific CD8^+^ T cells undergo apoptosis after interacting with macrophages in the liver, also confirming this.^49^ Antioxidant treatment and enzymatic blockade of ROS production effectively prevent the elevated FASL expression, and thus prevent CD8^+^ T cell exhaustion. Similarly, the blockade of TRAIL and NKG2D pathways demonstrates significant improvements in HBV‐specific T cell function.^48^

### 2.6 Amino acid metabolism is critical for CD8^+^ T cell protective roles

**FIGURE 3:**
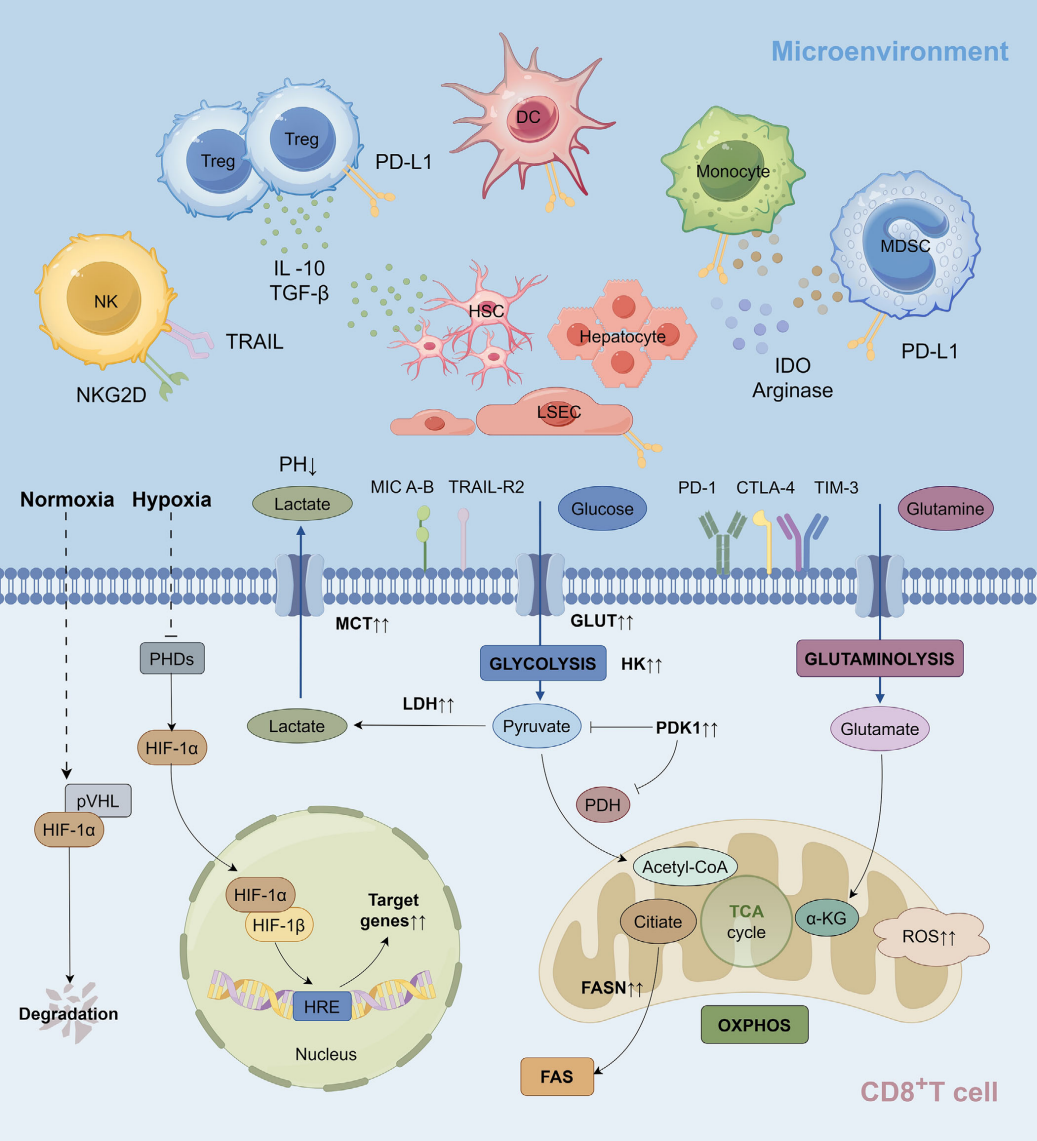
Related cells in liver anoxic microenvironment promote metabolic reprogramming of CD8^+^ T cells. The effect of related cells on CD8^+^ T cells in the liver microenvironment under hypoxia (upper part) and the corresponding metabolic reprogramming of CD8^+^ T cells (lower part). The expression of co‐inhibitory ligand PD‐L1 in different immunosuppressive cell populations such as Tregs, DCs, MDSCs and monocytes was up‐regulated under hepatic hypoxia, and the apoptotic signals induced by TRAIL and NKG2D receptors were increased on NK cells. A variety of cells, including HSCs, LSECs and hepatocytes, promote metabolic reprogramming of CD8^+^ T cells through a variety of mechanisms. In addition to producing soluble factors such as IDO and arginase, they can also promote CD8^+^ T cell exhaustion by expressing anti‐inflammatory cytokines such as IL‐10 and TGF‐β. In the cell, under normal oxygen conditions, HIF‐1α interacts with pVHL, triggering HIF‐1α degradation after ubiquitin ligase activity. In contrast, under hypoxia conditions, HIF‐1α enters the nucleus and binds to HIF‐1β protein, promoting the binding of DNA on HRE and playing a transcriptional activation role. Key glycolytic enzymes including LDH, PDK and HK are up‐regulated, which inhibits mitochondrial oxygen consumption. The up‐regulation of key metabolic enzymes such as GLUTs and LDH increases the conversion of glucose into lactate by glycolysis. PDK1 inhibits PDH, further prevents pyruvate oxidation in mitochondria, guides pyruvate to undergo glycolysis and promotes the reduction of mitochondrial OXPHOS. HIF‐1α also enhances the expression of lactate efflux transporter MCT, and the excretion of excess lactate leads to a decrease in extracellular PH. In addition to this, glutamine metabolism is also enhanced to stimulate the biosynthesis of fatty acids and amino acids, which in turn produces energy. NK, natural killer cell; Treg, regulatory T cell; DC, dendritic cell; MDSC, myeloid‐derived suppressor cell; HSC, hepatic stellate cell; LSEC, liver sinusoidal endothelial cell; NKG2D, natural killer group 2 member D; TRAIL, TNF‐related apoptosis‐inducing ligand; IL‐10, interleukin‐10; TGF‐β, transforming growth factor‐β; PD‐L1, programmed death ligand‐1; IDO, indoleamine 2,3‐dioxygenase; MIC A‐B, MHC class I chain‐related proteins A‐B; TRAIL‐R2, TNF‐related apoptosis‐inducing ligand‐R2; PD‐1, programmed cell death protein‐1; CTLA‐4, cytotoxic T‐lymphocyte‐associated protein‐4; TIM‐3, T‐cell immunoglobulin mucin domain‐3; HIF, hypoxia‐inducible factor; pVHL, Von Hippel‐Lindau protein; HRE, hypoxia response element; PHDs, prolyl hydroxylase domains; MCT, monocarboxylate transporter; LDH, lactate dehydrogenase; GLUT, glucose transporter; HK, hexokinase; PDK1, pyruvate dehydrogenase kinase 1; PDH, pyruvate dehydrogenase; CoA, coenzyme A; TCA, tricarboxylic acid; α‐KG, α‐ketoglutarate; FASN, fatty acid synthase; FAS, fatty acid synthesis; OXPHOS, oxidative phosphorylation; ROS, reactive oxygen species.

In addition to the factors discussed above can impair CD8^+^ T cells, amino acid metabolism also regulates the immune responses of these cells within the liver microenvironment. Systemic tryptophan metabolic reprogramming, triggered by inflammation, is intricately controlled in viral hepatitis.^50^ A variety of liver infiltrating cells, including immune and non‐immune cells, can release indoleamine 2,3‐dioxygenase (IDO), a tryptophan‐degrading enzyme. IDO‐controlled tryptophan catabolism promotes immunosuppression. IDO's activity in tryptophan catabolism leads to tryptophan depletion and the generation of the immunomodulatory toxic metabolite l‐kynurenines, which interacts with ligand‐activated transcription factor aryl hydrocarbon receptor (AHR), drives up‐regulation of PD‐1 in CD8^+^ T cells, thereby limiting T cell proliferation and inducing T cell exhaustion.^51^ Another critical amino acid, l‐arginine, faces depletion, primarily mediated by the key soluble mediator arginase (Arg). This enzyme is released by damaged hepatocytes and other liver‐infiltrating cells, the expression of several key enzymes in l‐arginine metabolism changes rapidly and significantly after inflammation, sepsis or injury.^52^ In CHB patients with elevated HBV replication levels, contributing to arginine depletion. The resultant arginine deficiency leads to T cell arrest in the G0/G1 phase, accompanied by the down‐regulation of the CD3ζ chain, further suppressing T cell proliferation. These immunosuppressive enzymes, secreted by monocytes and MDSCs, inhibit functional T cell proliferation and consume metabolites needed for maintenance.^53^ Monocyte MDSC (mMDSC) is differentially up‐regulated in HBeAg‐positive CHB patients, and HBeAg has also been shown to trigger mMDSC amplification, resulting in IDO‐mediated in vitro CD8^+^ T cell suppression.^54^ In addition, necrotic inflammation in the liver may also exacerbate the lytic release of these enzymes by hepatocytes, which further inhibits CD8^+^ T cells. Other cell populations, such as DCs, liver sinusoidal endothelial cells (LSECs) and hepatic stellate cells (HSCs), also promote T cell tolerance in the liver through a variety of mechanisms.^55^ In addition to producing IFN‐γ‐dependent soluble factors such as IDO and Arg,^56^ CD8^+^ T cell function is inhibited by regulating the expression of cell killing ligands such as FASL and TRAIL.^57^ In addition, these cells can also cause depletion of CD8^+^ T cells in a positive feedback loop of IL‐10 and TGF‐β1 by expressing anti‐inflammatory cytokines such as IL‐10 and TGF‐β, as well as up‐regulating co‐suppressor ligands, especially PD‐L1^58^, ^59^, ^60^ (Figure 3).

### 2.7 TOX links CD8^+^ T cell depletion in chronic HBV infection

Thymocyte Selection‐Associated High Mobility Group Box Protein (TOX), a nuclear DNA‐binding protein, plays a crucial role in lymphocyte development, as documented in numerous literatures. The up‐regulation of TOX expression emerges as a pivotal characteristic in the depletion of CD8^+^ T cells during persistent HBV infection. It facilitates the depletion phenotype of CD8^+^ T cells following chronic antigenic stimulation, proving to be a key factor in the normal progression of T cell dysfunction and the maintenance of depleted T cells during chronic infection. Additionally, TOX is instrumental in preventing overstimulation and activation of CD8^+^ T cells, averting cell death and limiting pathology.^61^, ^62^

Expressed by most cycling‐effect memory CD8^+^ T cell subsets, TOX is induced by calcineurin and nuclear factor of activated T cells 2 (NFAT2), operating in a feedforward cycle. It becomes independent of calcineurin and persists in depleted T cells, an essential component in T cell exhaustion. While TOX is not exclusively associated with exhaustion, its expression determines the characteristics of lytic genes and proteins, proving essential for the formation of effector T cells and memory T cells.^63^, ^64^ Furthermore, TOX promotes the persistence of antiviral CD8^+^ T cells and is necessary for progenitor CD8^+^ T cell programming. Consequently, long‐term immunity against chronic viral infections necessitates unique transcriptional and epigenetic programs associated with the transcription factor TOX. Intriguingly, besides maintaining the positive effects of antigen‐specific CD8^+^ T cells during persistent infection, TOX directly participates in the gene regulation of CD8^+^ T cell metabolism. Up‐regulation of TOX results in increased expression of genes involved in hypoxia response and down‐regulation of genes regulating OXPHOS, mammalian target of rapamycin (mTOR) signalling, IFN‐α response and DNA repair.^65^

Following TOX's identification as a major regulator of CD8^+^ T cell depletion, studies have explored its role in HBV‐specific CD8^+^ T cell dysfunction across different clinical stages of infection. The results showed that HBV‐specific CD8^+^ T cells TOX expression was closely associated with chronic antigen stimulation, viral load and T cell dysfunction. In chronic but non‐self‐limiting acute HBV infection, TOX expression in HBV‐specific CD8^+^ T cells persists even after spontaneous or treatment‐mediated viral control, this underscores TOX as a biomarker for dysfunctional virus‐specific CD8^+^ T cells in the context of persistent HBV infection and highlights its potential utility in guiding immunotherapy approaches.^66^

TOX has been shown to be a central regulator of depleted T cells in mice and is essential for effector and memory cell formation, but it is essential for exhaustion.^63^ Interestingly, follow‐up experiments found that compared with virus‐specific CD8^+^ T cells such as cytomegalovirus, Epstein–Barr virus and influenza virus, TOX expression in HBV‐specific CD8^+^ T cells was not correlated with terminal differentiation/senescence, indicating that the TOX expression in HBV‐specific CD8^+^ T cells was not correlated with the differentiation stage, as explained by the authors. Although TOX was equally highly expressed in the four virus‐specific T cells, the results varied because the transcription factor networks of the various virus‐specific CD8^+^ T cells differed. However, in CMV‐specific, EBV‐specific and FLU‐specific CD8^+^ T cells, neither amplification factor, cytokine production, nor degranulation were associated with TOX expression, suggesting that TOX only marks dysfunctional CD8^+^ T cells in persistent infections such as HBV infection, and future studies may be needed to demonstrate the complex epigenetic re‐modelling that occurs in depleted HBV‐specific CD8^+^ T cells.^66^

### 2.8 CD8^+^ T cell‐based immunotherapy for HBV infection

**FIGURE 4:**
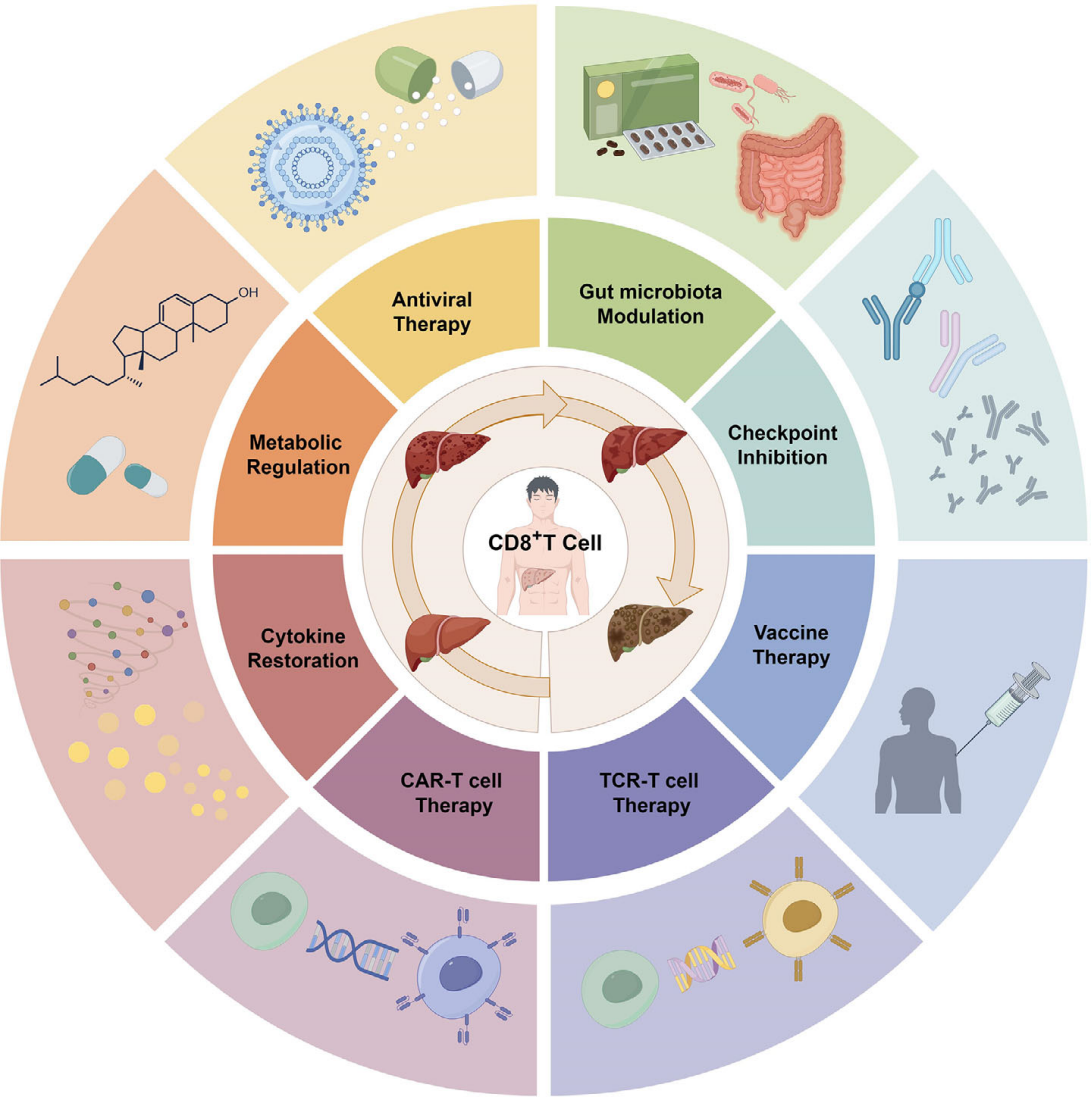
Therapeutic interventions based on CD8^+^ T cells, spanning from HBV infection to HCC, encompass diverse strategies for mitigating CD8^+^ T cell depletion during the progression from HBV to HCC. These strategies include antiviral therapy, metabolic and cytokine regulation, ICIs, vaccine prevention and T cell genetic engineering technology, along with emerging therapies focused on intestinal flora regulation. Some of these approaches have the potential to intercept the onset of HBV infection or HCC at its source, others may impede the further advancement from HBV to HCC, and some may even reverse the progression of established HCC. CAR‐T cell, chimeric antigen receptor engineered T‐cell; TCR‐T cell, T cell receptor engineered T‐cell.

Overall, CD8^+^ T cells undergo significant alterations during HBV infection, leading to weakened antigen‐handling capabilities in a hypoxic environment – a phenomenon termed cell exhaustion or dysfunction. Depleted HBV‐specific CD8^+^ T cells exhibit distinct features, including multiple inhibitory receptors, transcriptional reprogramming, extensive metabolic changes, effector dysfunction and a shift toward a memory‐like phenotype. Consequently, these changes have spurred the development of promising immunotherapeutic strategies for reinstating HBV‐specific immunity. These include immune checkpoint inhibitors (ICIs), metabolic T‐cell targeting therapy, therapeutic T‐cell vaccination and autologous T‐cell engineering involving chimeric antigen receptor (CAR)‐T cells and TCR‐engineered T (TCR‐T) cells (Figure 4).

#### 2.8.1 Immune checkpoint blockade

Up‐regulation of several inhibitory signalling pathways in HBV‐specific CD8^+^ T cells during chronic infection is believed to contribute to virus persistence. A promising therapeutic approach involves blocking inhibitory receptors to restore the function of depleted CD8^+^ T cells. Numerous studies have demonstrated that inhibiting the PD‐1/PD‐L1 interaction not only enhances CD8^+^ T cell proliferation but also augments the antiviral activity of peripheral HBV‐specific CD8^+^ T cells.^67^ However, solely blocking PD‐1/PD‐L1 is insufficient to completely reverse the immune function impairment induced by HBV infection. To enhance efficacy further, PD‐1/PD‐L1 inhibitors have been combined with other regulatory pathway interventions.^68^ This combined approach variably amplifies HBV‐specific CD8^+^ T cells, contributing to the restoration of CD8^+^ T cell response.

#### 2.8.2 Metabolism targeting therapy

Immune cell metabolism profoundly influences the immune response, and numerous studies have focused on the interdependence between T cell metabolism and function. Targeting T cell metabolism presents a promising therapeutic direction for disease treatment. In chronic HBV infection, depleted HBV‐specific CD8^+^ T cells display a significant increase in depolarised mitochondria and ROS production. This is accompanied by a marked down‐regulation of various cellular processes, highlighting mitochondrial dysfunction. Consequently, mitochondria emerge as viable targets for novel CHB therapies. ROS are central to T cell exhaustion, and mitochondria‐targeted antioxidants effectively enhance mitochondrial function and antiviral CD8^+^ T cell activity. These antioxidants neutralise excess ROS, improving mitochondrial depolarisation, electron transport chain protein expression and HBV‐specific CD8^+^ T cell cytokine production.^69^ Meanwhile, ROS overproduction disrupts the protein degradation mechanism in HBV‐specific CD8^+^ T cells, presenting another therapeutic target for CHB. In vitro exposure to natural polyphenols, such as resveratrol and oleuropein, rectifies some dysregulated intracellular pathways and bolsters antiviral CD8^+^ T cell function. The combined use of mitochondria‐targeted antioxidants and natural polyphenols offers a promising strategy for immune reconstitution in chronic HBV infection.^33^

#### 2.8.3 Cytokine restoration therapy

Moreover, the accumulation of inhibitory cytokine IL‐10 impairs the proliferation and cytokine secretion abilities of CD8^+^ T cells. IL‐10R signalling in antigen‐specific T cells makes them dysfunctional, and inhibition of IL‐10Ra can restore antiviral immunity. Blocking IL‐10 restores CD8^+^ T cell functionality and aids in viral elimination, reversing T cell exhaustion. Notably, IL‐10Ra blocking also restored the functional phenotype of T cells in vaccinated cirrhotic patients.^70^ IL‐12 enhances HBV‐specific CD8^+^ T cell effector cytokine production in response to antigen stimulation. It achieves this by decreasing the percentage of depolarised mitochondria, augmenting mitochondrial potential and reducing glycolytic dependence.^22^ IL‐12‐mediated restoration involves the down‐regulation of the inhibitory receptor PD‐1, an increase in the transcription factor T‐box transcription factor, and a reduction in the pro‐apoptotic molecule Bim, which mediates premature depletion of HBV‐specific CD8^+^ T cells. The combination of IL‐12 with PD‐1 pathway inhibitors further enhances CD8^+^ T cell function in most patients.^71^ Furthermore, administering recombinant IL‐2 to HBV transgenic mice has shown that virus‐specific CD8^+^ T cells regain their proliferative and differentiation capabilities into IFN‐γ‐producing cells, indicating that IL‐2‐based strategies are effective for chronic HBV infection therapy.^72^

#### 2.8.4 Therapeutic T cell vaccination

The spontaneous regression of HBV infection, often associated with immune reestablishment, suggests that stimulating HBV‐specific T cell immunity via therapeutic vaccination is a viable approach to overcome immune tolerance in chronic cases.^73^ Therapeutic vaccination, a burgeoning research area, has seen several formulations tested in both animal models and humans. These include protein or peptide‐based, DNA‐based and viral vector‐based vaccines, representing diverse classes of immunogens.

Clinical trials have evaluated numerous vaccine therapies for HBV infection, focusing on enhancing therapeutic vaccination efficacy through the addition of other immunomodulators, such as checkpoint inhibitors and metabolic regulators.^74^, ^75^, ^76^ Despite these efforts, a consistently successful therapeutic vaccine capable of breaking chronic HBV infection has yet to be developed.^77^ This underscores the need for continued research and development in therapeutic T‐cell vaccines targeting HBV infection, aiming to achieve more effective outcomes.

#### 2.8.5 Adoptive transfer of genetically engineered T cells

During HBV infection, CD8^+^ T cells often become dysfunctional. Immunotherapy, particularly the use of newly generated TCR‐T cells or CAR‐T cells, shows promise in replacing these dysfunctional cells. Although most adoptive transfers of genetically engineered T cell therapies have focused on malignant tumours, there is increasing clinical evidence supporting their potential effectiveness in enhancing HBV‐specific T cell responses. Specifically, in chronic HBV infection, adoptive transfer of engineered antigen‐specific T lymphocytes has demonstrated efficacy in clearing the virus.^78^

Efforts to redirect the specificity of existing T cells in HBV infection involve transferring the HBV‐TCR gene. This process has been tested in HBV transgenic mice and patients with HBV^+^HCC relapse. It involves isolating, amplifying and activating circulating T lymphocytes from CHB patients in vitro. These cells are then engineered using viral vectors encoding HBV‐specific TCRs to specifically target HBV. The modified, fully functional T cells are subsequently reintroduced into patients with chronic infection. These engineered lymphocytes have shown the ability to recognise and lyse HBV‐infected liver cells.^79^, ^80^ In a different approach using chimeric immunodeficient mice with persistent HBV infection, hepatitis B surface antigen (HBsAg)‐CAR‐T cells have been studied. These CAR‐T cells have demonstrated the capability to reduce HBV‐DNA and HBsAg levels, indicating their anti‐HBV activity in preclinical models.^81^ Altogether, genetically engineered T cell adoptive transfer therapy represents a promising immunotherapy approach for antiviral use. However, despite increasing research into the challenges and potential of T cell engineering therapy, it remains technically complex, particularly in the context of chronic HBV infection.

Overall, CD8^+^ T cells play an important role in HBV virus control and CHB disease progression, but the number of CD8^+^ T cells in the liver microenvironment is reduced and their function is impaired. The progressive hypoxia of liver microenvironment during HBV infection directly or indirectly changes the activity, proliferation and cytokine secretion of CD8^+^ T cells, and is accompanied by corresponding metabolic reprogramming, which is manifested as increased cellular glycolysis, glutamine metabolism and FAS, decreased OXPHOS, mitochondrial dysfunction and abnormal amino acid metabolism. An acidic microenvironment that promotes extracellular lactate accumulation. In addition, relevant cells in the liver microenvironment also have effects on CD8^+^ T cells, including binding to the up‐regulated inhibitory receptors of CD8^+^ T cells and increasing apoptosis signalling, which together promote CD8^+^ T cell depletion. In conclusion, in HBV infection and CHB, there is a complex interrelationship between the antiviral efficacy of CD8^+^ T cells and multiple mechanisms. Some strategies for HBV infection and CHB treatment have been derived, including ICIs, metabolic targeted therapy, therapeutic T cell inoculation, adoptive transfer of genetically engineered CD8^+^ T cells and so on (Table 1). Because of the variety of ways in which the HBV evades the immune system, the level and quality of the resulting immunotherapy has remained elusive, although it is widely accepted that CD8^+^ T cells play a crucial role in antiviral immunity in CHB patients. Therefore, one drug or immunotherapy is unlikely to produce a good response in most CHB patients, which may require a combination treatment regimen that includes direct‐acting drugs that target different stages of the viral life cycle, as well as stimulation of the antiviral immune response. It is worth noting that TOX has a potential role to play in guiding immunotherapy approaches as a biomarker of dysfunctional virus‐specific CD8^+^ T cells in the context of persistent HBV infection.

**TABLE 1:** Therapeutic interventions for CHB based on CD8^+^ T cells.

Therapeutic intervention	Mechanism of action and efficacy	Specific treatment drugs and methods	Clinical trial number and references
Immune checkpoint blockade	Blocking immunosuppressive pathways such as PD‐1/PD‐L1 reversed HBV‐specific CD8^+^ T cell exhaustion.	Cetrelimab	NCT05242445
		Nivolumab	NCT04638439
		HLX10	NCT04133259
		ASC22	NCT04465890
		RO7191863	NCT04225715
Metabolism targeting therapy	Mitochondria‐targeting antioxidants enhance mitochondrial function and antiviral CD8^+^ T cell activity.	Natural polyphenol	^33^
Cytokinesis restoration therapy	Increased HBV‐specific CD8^+^ T cell effector function by modulating cytokines.	IL‐12	^22^
		IL‐2	^72^
Therapeutic T cell vaccination	Inducing HBV‐specific CD8^+^ T cell responses or generating new CD8^+^ T cells to expand effector function.	TG1050	NCT02428400^82^
		HBsAg‐HBIG (YIC)	ChiCTR‐TRC‐11003189^76^
		Nasvac	NCT01374308^83^
		GS‐4774	NCT01779505^84^
			NCT01943799^75^
Genetically engineered T cells	TCR redirects T cells that have transferred the HBV‐TCR gene into the body to specifically target HBV.	TCR‐T cells	^80^, ^85^
	HBsAg‐CAR T cells with the ability to reduce HBV‐DNA and HBsAg levels enter the body to reduce HBV activity.	CAR‐T cells	^79^, ^81^

Abbreviations: CAR, chimeric antigen receptor; CAR‐T cells, chimeric antigen receptor engineered T‐cells; CHB, chronic hepatitis B; HBsAg, hepatitis B surface antigen; HBV, hepatitis B virus; IL‐12, interleukin‐12; IL‐2, interleukin‐2; PD‐1, programmed cell death protein‐1; PD‐L1, programmed death ligand‐1; TCR, T cell receptor; TCR‐T cells, T cell receptor engineered T‐cells.

## 3 CD8^+^ T CELL IMMUNITY IN LIVER FIBROSIS AND CIRRHOSIS

### 3.1 The microenvironment of liver fibrosis/cirrhosis weakens CD8^+^ T cell responses

Chronic HBV infection can lead to repeated liver injury, which may result in chronic inflammation, matrix deposition and angiogenesis, culminating in progressive fibrosis.^86^ This fibrotic response is characterised by an excessive accumulation of collagen, impairing normal liver regeneration and increasing the risk of liver failure. While early‐stage liver fibrosis is reversible, advanced fibrosis can progress to cirrhosis, where liver cells are replaced by scar tissue and liver stellate cells produce excessive collagen. This environment is conducive to the development of liver cancer.^87^, ^88^

There is a bidirectional relationship between cirrhosis and immune function. Immune‐mediated inflammatory mechanisms contribute to the pathogenesis of cirrhosis, and conversely, cirrhosis leads to dysregulated immune impairment and immune cell activation.^89^ In HBV‐related fibrosis, CD8^+^ T cell cytotoxicity, critical for viral clearance, can directly mediate HSCs activation and fibrogenesis. CD8^+^ T cells attract HSCs in a chemokine receptor 5 (CCR5)‐dependent manner and induce the activated HSCs to FASL‐FAS‐mediated apoptosis, thus promoting the regression of liver fibrosis. In short, liver CD8^+^ T cells play an important role in fibrosis regression.^90^

The variation in CD8^+^ T cell numbers in the cirrhotic liver microenvironment is a subject of debate. For instance, an analysis of T cell distribution in cirrhotic tissues from HCV and HBV patients, as well as healthy controls, showed a decrease in the CD4/CD8 ratio in cirrhotic tissue, but no significant difference in CD8^+^ T cells in liver.^91^ In contrast, recent report, focusing on cirrhosis caused by non‐alcoholic fatty liver disease (NAFLD), alcohol and primary biliary cirrhosis, has observed a decrease in the proportion of CD8^+^ T cells and an increase in CD4^+^ T cells.^92^ These discrepancies could be attributed to the differing aetiologies of cirrhosis in the studies, underscoring that changes in the T cell population might depend on the underlying cause of the fibrotic process.

### 3.2 Hypoxia likely impairs CD8^+^ T cell function in liver fibrosis/cirrhosis

In the liver, physiological oxygen gradients are crucial for metabolic zoning. Pathologically, hypoxia is a significant factor in liver diseases. This is evident in the liver microenvironment during HBV infection and liver fibrosis, where hypoxia is both a consequence and a promoter of liver fibrosis.^93^

Hypoxia influences liver fibrosis by up‐regulating the expression of HIF‐1α and NF‐κB. This activation stimulates HSCs, induces angiogenesis, epithelial–mesenchymal transition, mediates chronic inflammation and causes genetic modifications, all contributing to fibrosis development. Hepatic sinusoidal capillarisation, another inducer of hypoxia, can further aggravate liver fibrosis and lead to cirrhosis, underlining the close relationship between hypoxia and the progression of liver fibrosis and cirrhosis.^93^ Currently, research on the impact of the liver microenvironment on CD8^+^ T cells in liver fibrosis and cirrhosis is limited. However, given the hypoxic characteristics of the liver microenvironment during HBV infection, it is plausible that CD8^+^ T cells undergo similar changes during liver fibrosis and cirrhosis. Thus, it is reasonable to hypothesise that the alterations observed in CD8^+^ T cells in the liver microenvironment during fibrosis and cirrhosis are largely influenced by hypoxia, akin to the changes seen during HBV infection.

### 3.3 Metabolic reprogramming contributes to CD8^+^ T cell dysfunction

In chronic liver disease and liver fibrosis, inflammation is a central event, with hypoxia being a critical microenvironmental factor. Hypoxia mediates the accumulation of ROS and inflammatory cytokines in various liver‐associated cells, such as KCs, white blood cells, lymphocytes and NK cells, following liver injury. This process is intimately linked to the onset and progression of chronic inflammation.^93^

During liver fibrosis, hypoxia‐induced generation of free radical oxygen species stimulates ROS production by cytosolic nicotinamide adenine dinucleotide phosphate oxidase. ROS also arises from other types of liver damage, contributing to liver fibrosis. These increased free radicals, coupled with hypoxia, activate NF‐κB or act through the mitogen‐activated protein kinase pathway, elevating the expression of inflammation‐related factors and chemokines.^93^ As observed in HBV infection, elevated ROS levels within immune cells can lead to overactivation of the inflammatory response, causing tissue damage and cell necrosis. Thus, high ROS levels in liver fibrosis/cirrhosis may directly contribute to CD8^+^ T cell dysfunction and negatively impact cell viability.

Similarly, liver fibrosis, like the hepatitis phase, can lead to an accumulation of IL‐10‐secreting cells in the liver. This increase in IL‐10 secretion impairs the functionality of CD8^+^ T cells and may contribute to the development of HCC.^92^ Given the role of hypoxia in inducing IL‐10 accumulation, which in turn diminishes CD8^+^ T cell proliferation and cytokine secretion, it is plausible that these processes share a common mechanism.^94^

### 3.4 Immune checkpoints up‐regulated in CD8^+^ T cells in liver fibrosis/cirrhosis

CD8^+^ T cells in patients with liver cirrhosis exhibit phenotypic, functional and transcriptional characteristics of cirrhose‐associated immune dysfunction. Particularly, CD8^+^ T cells in cirrhotic patients showed a notable enrichment of human leukocyte antigen DR (HLA‐DR) ^+^CD8^+^ phenotype in their livers. These cells exhibit high expression levels of immune checkpoints, including TIM‐3, CTLA‐4 and PD‐1. Functionally, HLA‐DR^+^CD8^+^ T cells demonstrate reduced proliferation of peripheral blood mononuclear cells compared with their HLA‐DR^−^ counterparts and induce phenotypic and functional impairments in monocytes and neutrophils in vitro. Furthermore, in HLA‐DR^+^CD8^+^ T cells from cirrhotic patients, there is a significant down‐regulation of genes involved in signalling pathways and cytokine production. This particular subgroup of CD8^+^ T cells in cirrhotic patients may influence their susceptibility to infections and is associated with a poorer disease prognosis.^95^

### 3.5 CD8^+^ T cell‐based immunotherapy for liver fibrosis/cirrhosis

Liver cirrhosis, often precipitated by HBV infection, disrupts the immune system's environmental homeostasis. Without effective treatment, HBV infection can progress to liver fibrosis and eventually cirrhosis, underscoring the close relationship between liver inflammation and fibrosis.^96^ Consequently, immunotherapy for liver cirrhosis primarily focuses on the aetiology of HBV infection. Since specific mediators driving changes in dysfunctional CD8^+^ T cell subsets in cirrhosis need further investigation, and their clinical relevance is yet to be fully understood, current immunotherapy strategies for cirrhosis predominantly target cells other than CD8^+^ T cells, such as macrophages.^97^, ^98^ However, considering the observed enrichment of dysfunctional CD8^+^ T cell subsets in cirrhotic patients,^95^ it is plausible to speculate that CD8^+^ T cell‐based therapy (Figure 4), when applied early in HBV‐induced liver fibrosis and then cirrhosis, might still be effective for disease control.

### 3.6 Combination of gut microbiota and CD8^+^ T cells indicates new therapy direction for liver fibrosis/cirrhosis

In recent years, gut microbiota regulation therapy has emerged as a novel treatment method, gaining increasing attention for its role in liver diseases. The liver, connected to the gut through the porta hepatis, biliary secretion system and portal vein,^99^ is significantly influenced by the gut microbiota. There is growing evidence that the gut microbiota plays a crucial role in the formation, pathogenesis^100^, ^101^ and therapeutic response of liver diseases, including NAFLD, fibrosis, cirrhosis and HCC.^102^, ^103^, ^104^ The regulation of the gut microbiota is being recognised as a potential therapeutic target due to its significant role in liver disease progression.

Alterations in the gut microbiota are a risk factor for liver disease. Prior to the development of HCC, the gut microbiota composition of patients with HBV related cirrhosis was significantly changed,^105^ with some local taxa (such as *Lachnospiraceae*, *Ruminococcaceae* and *Clostridiales* XIV) decrease while some pathogenic taxa (such as *Enterococcaceae*, *Staphylococcaceae* and *Enterobacteriaceae*) increase, and this imbalance increases as the disease progresses.^106^, ^107^ In addition, genetic and metagenomic species richness is significantly reduced in patients with cirrhosis, and this loss of richness correlates with disease stage and prognosis.^108^ In conclusion, regardless of the cause of cirrhosis, there is a serious imbalance in the presence of gut microbiota, especially in the advanced stages of the disease.^109^ The disruption of peripheral immune homeostasis by an imbalanced gut microbiota entering the liver can shape the hepatitis microenvironment, leading to the progression of HBV‐associated hepatitis to fibrosis, cirrhosis and eventually HCC.^99^, ^110^, ^111^

The gut microbiota can reprogram immunity by participating in the regulation of innate and/or adaptive immune cells. Changes in the gut microbiome can also have an impact on CD8^+^ T cells. Studies have shown that specific gut microbiota can regulate CD8^+^ T cells. For example, *Bifidobacterium*, *Enterococcus*, *Faecalibacterium*, *Ruminococcus* and *Clostridiales* can promote CD8^+^ T cells to infiltrate tumour tissue, and *Phyla Firmicutes* and *Actinobacteria* can enhance the activation of CD56^+^CD8^+^ T cells.^112^ In cirrhosis, disruptions in gut microbiota balance, bacterial overgrowth and increased intestinal wall permeability compromise protective mechanisms. This leads to pathological bacterial translocation and heightened uptake of endotoxins, which reach the liver and mesenteric lymph nodes, activating immune cells and triggering the release of pro‐inflammatory cytokines like TNF‐α and IL‐8,^101^ including CD8^+^ T cells. Moreover, an increased bacterial presence in the liver tissues of cirrhotic patients prompts transcriptional changes, activating fibrous inflammatory pathways and pathways associated with cancer immunosuppression,^110^ this induces inflammation in both the liver and the systemic environment. In conclusion, the gut microbiome and its metabolites influence all aspects of CD8^+^ T cell development, differentiation and function.

Liver disease is linked to the gut microbiome, suggesting the possibility of treating cirrhosis and intervening early by controlling the gut microbiome. Manipulating the gut microbiota has potential utility in treating the hepatitis microenvironment, delaying the progression of cirrhosis and preventing cancer. Approaches such as dietary changes, probiotics or faecal microbiota transplantation (FMT) can be employed to promote the growth of beneficial bacteria, ameliorating dysbiosis and altering disease prognosis.^101^ Intestinal flora can regulate immune response and affect the efficacy of immunotherapy.^109^ The combination of intestinal microbiome therapies such as antibiotics, probiotics, FMT and bacterial genetic engineering with the therapeutic strategy of ICI provides new possibilities for the treatment of intestinal microbiota. In addition to modulating ICI immunotherapy, gut microbiota can also influence other cell‐based immunotherapies, such as adoptive transfer of T cell immunotherapy.^112^ This is expected to influence the development of chronic liver disease and health improvement in the future. Therefore, further research into the interactions between gut microbes and the host immune system is necessary to shed light on the pathogenesis of liver fibrosis/cirrhosis and open up new opportunities for immunotherapy or gut microbiome‐based therapies.

All in all, after chronic HBV infection progresses to the stage of liver fibrosis and cirrhosis, the liver microenvironment continues to maintain the characteristics of hypoxia, and hypoxia promotes the formation of liver fibrosis, which in turn aggravates hypoxia, forming a vicious circle of positive feedback. The rise of ROS stimulated by hypoxia may directly have a negative impact on the viability of CD8^+^ T cells, and the induced IL‐10 accumulation may also reduce the proliferation of CD8^+^ T cells and cytokine secretion. In addition, CD8^+^ T cell surface immune checkpoints continued to be up‐regulated. Immunotherapy of liver fibrosis and cirrhosis mainly focuses on the aetiology of HBV infection. According to the similar characteristics of chronic HBV infection and liver fibrosis, CD8^+^ T cell‐based therapy can still achieve therapeutic effect in the early stage of HBV‐induced liver fibrosis and cirrhosis. With the deepening of gut microbiota research in recent years, the treatment of liver fibrosis/cirrhosis has a new direction. Dietary changes, probiotics or FMT and other methods can improve the imbalance of gut microbiota, which has a positive effect on the treatment of liver fibrosis and cirrhosis.

## 4 ANTI‐TUMOUR IMMUNITY OF CD8^+^ T CELLS BECOMES EXHAUSTED IN HCC

### 4.1 Tumour microenvironment comprehensively impairs CD8^+^ T cell immunity

CD8^+^ T cell exhaustion, a phenomenon commonly observed during prolonged chronic infections and cancers in humans, may serve as a protective mechanism against immunopathology. However, it also limits the effective containment of pathogens and cancer.^113^, ^114^, ^115^ In the context of HBV infection, transformation of liver cells into HCC is primarily the consequence of chronic interactions between HBV and host liver cells. The potential mechanisms include not only the integration of HBV DNA, but may also include the long‐term expansion of virus HBx regulatory proteins and/or abnormal preS/S envelope proteins.^116^

The tumour microenvironment (TME) in HCC significantly influences tumour progression. It consists of malignant cells, different subpopulations of immunocytes, a complicated environment of cell factors and the extracellular matrix.^117^, ^118^, ^119^ Among them, different innate and adaptive immune cells influence the immune evasion of the tumour and the response to immunotherapy.^120^ Examples include MDSCs, one of the most common innate immune cells in HCC, which are highly immunosuppressive. MDSCs can express IDO, Arg1, TGF‐β, cyclooxygenase‐2 and IL‐10, which inhibit a variety of immunocytes, including T cells.^121^ Alternatively, abiotic characteristics of the TME, such as hypoxia, acidic pH, altered metabolites and a range of growth factors and immunosuppressive cytokines, shape both the tumour cell reservoir and impact the health and function of resident and tumour‐infiltrating immune cells.^122^, ^123^

CD8^+^ T cells, critical lymphocytes with anti‐tumour effects in the TME of HCC, produce perforin and other cytotoxins that target malignant cells while sparing normal ones.^124^ Studies using HBV or HCV‐related HCCs (scRNA‐seq) have revealed that CD8^+^ T cell clusters are more abundant in HBV or HCV‐related HCCs compared with non‐HBV/HCV‐related HCCs, with chronic HBV/HCV infection states associated with increased cytotoxic T cells (CTLs) exhaustion.^125^

The efficacy of CD8^+^ T cell‐mediated anti‐tumour immune response in HCC is hindered by several factors: the high expression of immunomodulatory molecules (e.g. PD‐1, PD‐L1, TIM‐3, LAG‐3, IDO1) in white blood cells or malignant cells, physicochemical imbalances in the microenvironment (like hypoxia and acidic pH), metabolic competition with malignant cells, and the lack of support from CD4^+^ T cells.^126^, ^127^ Additionally, CD8^+^ T cells from HCC tissues exhibited high levels of markers of exhaustion, reduced proliferation, decreased cellular activation and reduced generation of effector cell factors compared with CD8^+^ T cells from CHB tissues.^128^

Overall, CD8^+^ T cells, as the primary anti‐tumour cells in immunotherapy, can be activated by tumour‐associated antigens (TAAs) to specifically kill cancer cells, which determines the anti‐tumour effects considerably.^114^ As a consequence, an in‐depth understanding of the changes in TME in HBV^+^HCC and the mechanism by which it leads to CD8^+^ T cell exhaustion could offer an essential theory for the clinical application of immunotherapies with future applications.

### 4.2 Hypoxia depresses CD8^+^ T cell functions in HCC

Hypoxia is a critical characteristic of the TME in solid tumours, including HCC, and plays a key role in creating an immunosuppressive TME. This allows malignant cells to evade innate and adaptive immune defenses.^129^, ^130^ Practically, hypoxia not only facilitates the proliferation, migration and invasion of HCC cells, but also hastens malignant progression by affecting the crosstalk between stromal cells, tumour cells and immune cells in the TME.^131^ Intra‐tumoural hypoxia in HBV^+^HCC is linked to poor prognosis,^130^ partly due to its detrimental impact on CD8^+^ T cell functions.

Hypoxia induction is mediated by a major molecular mechanism through HIFs. In particular, HIF‐1/2α protein is highly expressed in human HCC tissues, with its expression correlating with poor clinical prognosis in HCC patients.^121^ HIF‐1α induces increased expression of triggering receptor expressed on myeloid cells‐1 (TREM‐1) on tumour‐associated macrophages (TAMs), impairing the cytotoxic function of CD8^+^ T cells and inducing CD8^+^ T cell apoptosis.^132^ Compositional expression of HIF‐1α also resulted in diminished immunostimulatory capacity of DCs, showing an increase in inhibitory mediators including IL10, inducible nitric oxide synthase and vascular endothelial growth factor (VEGF), which associated with a reduction in their ability to drive effector CD8^+^ T cell functions.^133^ Additionally, the targeting of HIF‐1α is considered a potential therapeutic approach in cancer treatment, as both HIF‐1α and its direct target, PD‐L1, have been linked to poor prognosis in HCC.^134^ Interestingly, under hypoxic conditions, CD8^+^ T cells can differentiate more efficiently into CTLs.^135^ It is demonstrated that HIF‐1α is crucial in regulating CD8^+^ T cell effector responses in the TME, influencing their glycolytic metabolism, migration and effector functions.^136^ For example, CD27, as a co‐stimulatory molecule on T cells, promotes the proliferation of CD8^+^ T cells and their differentiation into effector cells upon binding with its ligand CD70,^137^ playing a crucial role in T cell activation. However, due to the increased glucose consumption mediated by HIF‐1α signalling, a low‐glucose microenvironment is established, leading to the suppression of CD27 transcription, thereby affecting the cytotoxic function of CD8^+^ T cells. Notably, HIF‐2α, in particular, has been shown to enhance the anti‐tumour cytotoxicity of CD8^+^ T cells when ectopically expressed, in contrast to HIF‐1α.^138^ In addition, HCC treatments such as arterial chemoembolisation and the anti‐tumour drug tyrosine kinase inhibitors, while slowing down tumour growth, also unintentionally exacerbate hypoxia. Remarkably, hypoxia induces metabolic reprogramming through HIFs, which can lead to resistance to the current HCC treatments and lead to poor HCC outcomes.^139^ Therefore, overcoming drug resistance in hypoxic HCC is possible to improve patients' quality of life.

As mentioned above, HIFs have an essential role in the regulation of hypoxic TME and HCC development. Currently, inhibitors targeting HIFs or their signalling pathways show promising clinical potential in the treatment of HCC, such as CT‐707 (a potent inhibitor of YAP signalling). CT‐707 disrupts the hypoxia‐induced IGF1R‐YAP axis under hypoxia, making it a hopeful therapeutic option for HCC.^140^ However, our understanding of tumour hypoxia in HCC is still in its infancy, so we need to continue to delve deeper into the influence of hypoxia on the TME of HCC and on CD8^+^ T cells, and work to translate this into clinical applications to broaden new therapeutic strategies against HCC.

### 4.3 Metabolic reprogramming and lactate microenvironment regulate CD8^+^ T cell responses

The ‘Warburg effect’, where tumour cells predominantly undergo aerobic glycolysis instead of the more energy‐efficient mitochondrial phosphorylation for energy production, significantly contributes to the acidic extracellular TME in solid tumours.^141^, ^142^ This metabolic shift enhances glucose uptake and fermentation into lactate.^143^ Hypoxic TME can further exacerbate this effect by up‐regulating genes encoding glucose transporters and glycolytic enzymes, such as LDH, leading to increased lactate production and secretion by tumour cells, thus lowering the pH of the TME.^144^ Li et al.^145^ identified six key lactate metabolism‐related genes (LMRGs), including FKTN, PET117, PDSS1, PUS1, RARS1 and RNASEH1, through database screening, which have significant clinical value in independently predicting the prognosis of HCC patients. They found that patients in the LMRGs‐high group had overall and median survival that was notably shorter than in the LMRGs‐low group. Furthermore, the immune microenvironment in the LMRGs‐high group showed a state of suppression, with a greater infiltration of suppressive immune cells.

A decrease in extracellular pH within the TME profoundly impacts infiltrating immune cells. Accumulation of lactate induces differentiation of TAMs, Tregs and MDSCs stimulates their biological activity, which in turn leads to the secretion of immunosuppressive factors that suppress the immune response of T and NK cells, assisting in escaping tumour cells from immune surveillance and gaining unlimited growth potential.^144^ It reported that the cytotoxic activity of CD8^+^ T cells decreases in a pH‐dependent manner, with functional CD8^+^ T cell induction being markedly impaired under low pH conditions.^146^ Tumour‐derived lactate changes the entry of pyruvate into the TCA cycle, affecting CD8^+^ T cell metabolism and reducing their cytotoxicity. But, inhibiting PDH increases pyruvate carboxylase (PC) activity and succinate secretion, which can activate the succinate receptor 1 (SUCNR1) and enhance anti‐tumour cytotoxicity in CD8^+^ T cells.^147^

Additionally, hepatic mitoribosomal defects can shift hepatic glucose metabolism towards glycolytic flux and lactate synthesis, creating an unfavourable lactic acid microenvironment for CD8^+^ T cells. This environment is conducive to CD8^+^ T cell exhaustion and cancer progression.^148^ Interestingly, a study indicated that in highly glycolytic tumours, lactic acid down‐regulates PD‐1 expression in CD8^+^ T cells and induces PD‐1 expression in Tregs.^149^ Under high lactate conditions, on the other hand, the primary effect of PD‐1 blockade transitions from stimulating CD8^+^ T cells to suppressing them through Tregs, effectively converting the blockade's immunostimulatory effect into an immunosuppressive one.^149^, ^150^ Furthermore, high lactate levels in the TME can impede T‐cell lactate output, further disrupting CD8^+^ T cell metabolism and function.

To summarise, in HCC, the acidic TME caused by the ‘Warburg effect’ not only provides a survival advantage for tumour cells, but also impairs the anti‐tumour function of immune cells represented by CD8^+^ T cells to a certain extent. To target the metabolic pathway represents a practical strategy for enhancing the immunogenicity of tumours.

### 4.4 Amino acids in TME metabolically reprogram CD8^+^ T cell functions

Amino acids are crucial for protein synthesis, energy production and redox balance maintenance in mammals.^151^ Given the liver's central role in amino acid metabolism, alterations in amino acid and protein metabolism are significant in supporting the robust biosynthesis observed in HCC.^152^ In TME, tumour cells often consume nutrients, including glucose and amino acids, at a faster rate than infiltrating immune cells. This nutrient competition disrupts the availability of energy resources essential for the effector functions of immune cell.^153^ Herein, we will specifically focus on the impacts of three amino acids (glutamine, tryptophan and methionine) on CD8^+^ T cells within the context of HCC.

#### 4.4.1 Glutamine

Glutamine, the highest abundant amino acid in the blood, plays a critical role in energy production and anabolic processes, acting as a key secondary carbon source after glucose.^154^ In hypoxic conditions, much of the glucose is converted into lactate, resulting in increased reliance on glutamine carbon.^155^ HCC is particularly dependent on extracellular glutamine, showing signs of glutamine addiction.^156^ Glutamine metabolism is vital for both cancer cell proliferation and the activation of CD8^+^ T cells, which are responsible for targeting and killing cancer cells. ScRNA‐seq data suggest that disrupting glutamine metabolism in tumour cells and CD8^+^ T cells could enhance the anti‐tumour capacity of CD8^+^ T cell subsets.^124^

Glutamine blockade in the tumour mice was demonstrated to impair oxidation and glycolysis metabolism of tumour cells, contributing to hypoxia and reduction in nutrient consumption. Conversely, activated CD8^+^ T cells adapt to glutamine blockade by up‐regulation of acetate catabolism, resulting in the production of large amounts of acetyl CoA, which directly or indirectly energises the TCA cycle, the latter through increased PC activity in response of glucose deficiency.^157^ In vitro studies have shown that CD8^+^ T cells deprived of glutamine have diminished capacity in producing effector factors and increasing suppressor markers (e.g. PD‐1) expression. This lack of glutamine impairs CD8^+^ T cell functionality, leading to altered mitochondrial morphology, elevated ROS levels and apoptosis induction.^158^ Notably, our group's research has also revealed that glutamine deprivation can significantly induce immune exhaustion in another subset of CTLs known as γδ T cells.^159^ Therefore, it is reasonable to conclude that glutamine metabolism plays a crucial role in the immune functions of T cells. Glutamine regulates T cell development and function by modulating mTOR activation and O‐GlcNAcylation in effector T cells. It also serves as the primary carbon source for the production of the metabolite 2‐hydroxyglutaric acid, which influences the function and differentiation of effector T cells.^153^

#### 4.4.2 Tryptophan

Tryptophan, an essential amino acid acquired solely through diet, and its metabolites are critical for the growth and maintenance of various cells, including their role in the development of liver cancer.^160^ More than 90% of mammalian tryptophan is metabolised by the kynurenine (KYN) pathway. In this, tryptophan oxidation is the initial step of the KYN pathway and mainly catalysed from IDO1 and tryptophan 2,3‐dioxygenase (TDO).^161^ The synergistic effect on tryptophan consumption and KYN accumulation renders TME highly immune‐suppressant, which reduces multiplication of effector T cells and favours Tregs differentiation.^162^

IDO is widely distributed in a variety of cell types (both immune and non‐immune cells) that is regulated by factors from immune response, inflammation and cytokines.^163^ In HCC, IDO1 is responsible for T‐cell suppression and for tumour cells evading surveillance and clearance by the immune system.^164^ As an example, IDO1 can enhance peripheral immune tolerance to TAAs by promoting tumourigenesis and the development of tolerogenic antigen‐presenting cells (APCs). Following, overexpression of IDO1 in tolerant APCs inhibited the activity and proliferation of CD8^+^ T effector cells and NK cells, but indirectly induced MDSCs and Tregs through the KYN pathway.^165^ In the HCC xenograft mouse model, Abrine, an IDO1 inhibitor, can down‐regulate the accumulation of the metabolite KYN through the Janus kinase 1–signal transducer and activator of transcription 1 (JAK1–STAT1) signalling pathway and inhibit tumour growth by increasing the infiltration of CD8^+^ T cells and decreasing Tregs.^164^ However, high expression of IDO1 in HCC has also been shown to positively correlate with the number of CD8^+^ T cells, which indicates the presence of an anti‐tumour immune response, suggesting that IDO1 may be a favourable prognostic indicator for patients with HCC. Moreover, they found no significant difference in IDO1 expression on inflammatory cells between HBV^+^HCC and HBV^−^HCC, considered to be a result of the small number of HBV^−^HCC patients included in their study.^166^ This seems to contradict some of the findings,^167^, ^168^ so the relationship between IDO1 and HCC as well as CD8^+^ T cells needs to be further investigated.

TDO is primarily located in the liver for the regulation of the hepatic conversion of tryptophan.^163^ In HCC, TDO is highly expressed in tumour tissues and remarkably correlates with malignant phenotypic features such as tumour differentiation, tumour size and vascular invasion, with the possibility that it may be an efficient biomarker for the diagnosis and prognosis of HCC.^169^ Additionally, Greene et al.^170^ in triple‐negative breast cancer, have found that TDO inhibits CD8^+^ T cell activity, reducing their anti‐tumour efficacy and leading to a negative prognosis. Therefore, it is reasonable to speculate that TDO could similarly inhibit CD8^+^ T cell activity in the TME of HBV^+^HCC, potentially leading to poor patient outcomes. As for the other hand, although no TDO‐specific inhibitors have yet entered clinical trials, inhibition of TDO can hopefully indirectly restrain suppressive immunological effects of IDO1 through increasing whole‐body tryptophan content and restricting IDO1‐generated KYN to T lymphocytes, and perhaps it will be a potential direction of future drug development.^161^

AHR is an endogenous KYN receptor that is thought to be important in immunomodulation and tumour development.^171^, ^172^, ^173^ As an example, AHR would play an essential role in HBx‐mediated HBV mechanisms, with the potential to contribute towards the progression of HCC following HBV infection.^174^ In HCC, tumour cells can promote the expression of pro‐proliferative genes and inhibit cell cycle inhibitory molecules to promote proliferative capacity via the IDO1‐AHR‐β‐catenin pathway.^175^ Alternatively, binding of KYN to AHR increases PD‐1 expression on tumour‐specific CD8^+^ T cells and induces differentiation and activation of immunosuppressive Tregs and facilitates the recruitment of tolerogenic myeloid cells.^161^ Liu et al.^176^ discovered that AHR probably functions as a key transcription factor in regulating CD8^+^ T cell exhaustion, such that in exhausted CD8^+^ T cells, inhibition of AHR activity or knockdown of AHR leads to the down‐regulation of PD‐1, TIM‐3, LAG‐3 and CD39, whereas IFN‐γ and TNF are up‐regulated. Moreover, they also found that it is IL‐2 that activates the STAT5–5‐hydroxytryptophan–AHR pathway and triggers CD8^+^ T cell exhaustion in the TME.^176^ Inhibitors of the IDO1‐KYN‐AHR pathway have been shown to alleviate the negative effects of Carboxyamidotriazole, a chemotherapeutic agent, against CD8^+^ T cells and produce supplementary useful pre‐tumour immunological effects.^177^ To sum up, AHR overexpression sends suppressive signals to immunological cells through TME and help malignant cells escape the immunity system.

#### 4.4.3 Methionine

Methionine, an essential amino acid crucial for cell growth and development, plays a vital role in protein synthesis, DNA methylation and polyamine synthesis in mammals, with its primary metabolism occurring in the liver.^178^, ^179^ A characteristic observed in certain cancers, including HCC, is ‘methionine dependence’, making methionine restriction a potential strategy to limit cancer growth.^180^ Notably, methionine levels are significantly higher in liver cancer tissue compared with adjacent liver tissue, and there is a marked correlation between tumour volume and serum methionine levels, indicating that tumour tissue competes successfully for methionine.^181^

Methionine which is an essential sulphur‐containing amino acid is catabolised and recycled within the series of metabolic reactions known as the methionine cycle.^182^ S‐adenosyl‐l‐methionine (SAM), the initial product of the ‘methionine cycle’, is synthesised from methionine and ATP by methionine adenosyltransferases (MATs) and serves as the main methyl donor in mammalian cells, particularly in the liver.^183^ Integromics analysis has shown that SAM and its metabolite 5′‐methylthioadenosine (MTA) in HCC directly impact the cytoplasmic accessibility of CD8^+^ T cells, predisposing them to functional exhaustion in the TME. Additionally, reprogramming of the methionine recycling machinery, potentially selected during tumourigenesis through somatic copy number alterations of methionine metabolism genes, leads to SAM/MTA accumulation in HCC.^127^ Besides, recent studies revealed that blocking MAT2A‐dependent methionine catabolism can cause cellular senescence by inducing cell cycle arrest and DNA damage in HCC. Therefore, the inhibition of methionine catabolism could be used as an improved pro‐senescence strategy for the treatment of HCC in combination with senolytic agents.^184^

Of interest is the fact that the level of methionine obtained from the diet may have a significant influence on cellular methionine metabolism with the ability to influence tumour growth.^179^, ^182^ Malignant cells impair methionine metabolism of CD8^+^ T cells, causing a decrease in cellular methionine. This disruption causes a decrease in H3K79me2 (an active transcriptional histone mark) and defects in the STAT5 signalling pathway in CD8^+^ T cells, impairing their immune function.^185^ Additionally, solute carrier family 43 member 2 (SLC43A2), a methionine transporter, is associated with higher levels of CD8^+^ T cells, higher markers of T cell exhaustion and lower levels of naive CD8^+^ T cells, suggesting that SLC43A2 may regulate immune‐related genes, leading to CD8^+^ T cell exhaustion and impacting the TME and prognosis of HCC.^186^ Therefore, supplementation of methionine and inhibition of tumour SLC43A2 can normalise methionine metabolism in effector T cells and restore their function, enhancing anti‐tumour immunity in preclinical models.^185^ The supplementation of methionine additionally reduced the growth rate of HCC cells and induced the adenosine monophosphate‐activated protein kinase (AMPK) and mTOR pathways.^178^ Nevertheless, studies have also suggested that tumour growth can be suppressed by restricting methionine diets. In the mouse cancer model, methionine restriction diets rapidly and specifically altered methionine and sulphur metabolism and suppressed the growth of tumours.^187^ What is more, methionine‐restricted diets may reduce tumour growth and enhance anti‐tumour through growing the amount as well as cell toxicity of CD8^+^ T cells infiltrating the tumour.^188^ Consequently, further research is needed on whether methionine diets can actually improve the prognosis for HCC patients.

Altogether, over‐lapping metabolic reprogramming in both tumour with immune cells has been thought to be a major factor determining the anti‐tumour immunological responses of tumour.^189^ We reviewed here the relationship between metabolic reprogramming of glutamine, tryptophan and methionine and HCC as well as CD8^+^ T cells, but in reality, there are many other amino acid metabolisms that can shape the TME of HCC, such as arginine metabolism.^190^, ^191^ For instance, Arg1 is expressed by regulatory myeloid cells in the TME (e.g. MDSCs) and may function as a pro‐tumourigenic and T‐cell suppressor in the TME. In contrast, CD8^+^ Arg1‐specific T cells can specifically affect TME through targeting and reducing Arg1‐expressing tumour cells together with regulatory myeloid lineages.^192^ Alternatively, appropriate supplementation with amino acids that have immunomodulatory properties (e.g. glutamine) has a significant effect on the maintenance of a moderate immune response.^189^ Hence, continuing further study of the metabolic reprogramming of amino acids in the TME of HCC is crucial for the diagnosis and treatment of HCC.

### 4.5 Immune checkpoints critically exhaust CD8^+^ T cell immunity

Functionally compromised HBV‐specific CD8^+^ T cells have been found to exhibit high‐level expression of several suppressor receptors, including PD‐1, CTLA‐4, TIM‐3 and LAG‐3. The overexpression of these inhibitory receptors, commonly referred to as immune checkpoints, can suppress T cell activity and contribute to their functional exhaustion.^193^ However, research indicates that blocking the overexpression of these immune checkpoints can potentially restore the functionality of CD8^+^ T cells. For instance, glucose depletion by tumour cells can metabolically restrict CD8^+^ T cells and directly impair their effector functions, leading to tumour progression. However, the application of checkpoint blockade therapies might correct this metabolic imbalance.^194^ These therapies can act directly on tumour cells to re‐establish resource availability for CD8^+^ T cells. Among the immune checkpoints, two particularly noteworthy are PD‐1 and CTLA‐4, which will be the focus of further discussion.

#### 4.5.1 PD‐1

PD‐1 is a crucial immune checkpoint receptor extensively studied in clinical cancer immunotherapy. It acts as an inhibitory receptor modulating immune responses.^195^, ^196^ The overexpression of PD‐1 on CD8^+^ T cells is a key factor contributing to their exhaustion, leading to impaired effector function and proliferation.^115^ In the context of HCC, the HBV^+^HCC group has been reported to exhibit the highest frequency of cells with high PD‐1 expression, compared with HCV‐associated and NASH‐associated HCC groups, with the latter showing relatively low PD‐1 expression.^197^ CD8^+^ T cells within HCC specimens express varying levels of PD‐1. Notably, distinct subsets of PD‐1^high^CD8^+^ T cells in HCC can co‐express TIM‐3 and/or LAG‐3 and are capable of producing IFN‐γ and TNF, albeit at low levels, in response to anti‐CD3 stimulation. Moreover, the level of PD‐1 expression on CD8^+^ T cells in peripheral blood may serve as a potential biomarker for identifying tumour‐infiltrating CD8^+^ T cells in HCC patients, which express various immune checkpoint receptors.^198^

Further studies have shown that CD8^+^ T cells with high PD‐1 expression down‐regulate not only effector cytokines such as IFN‐γ, IL‐2, TNF‐α, IL‐4, IL‐17A and IL‐22, but also the cytotoxic degranulation marker CD107a. These cells also exhibit diminished capacity to kill HCC tumour cells, such as HCC‐LM3. Additionally, CD8^+^ T cells with high PD‐1 receptor expression share characteristics with tissue‐resident memory T cells, evidenced by their abnormal activation status and increased apoptotic potential.^199^ Exhausted CD8^+^ T cells in HCC due to chronic antigen‐TCR stimulation often overexpress TOX, a high‐mobility group‐associated protein in the nucleus. TOX hinders PD‐1 from undergoing lysosome‐mediated degradation and promotes the endocytosis cycling of PD‐1 at the cell surface, further contributing to the persistence of T cell exhaustion.^200^

#### 4.5.2 CTLA‐4

CTLA‐4, another inhibitory TCR, is primarily expressed on activated T cells and Tregs, playing a crucial role in the negative regulation of T‐cell immune responses.^201^, ^202^ Unlike PD‐1, CTLA‐4, present on mature T cells, can bind to its ligands CD80/86 on various types of APCs. This binding inhibits the immune response initiated by the interaction of T cell CD28 with CD80/86.^203^

In HCC tissues, exhaustion markers such as PD‐1, CTLA‐4, TIM‐3 and LAG‐3 are expressed at higher levels in CD8^+^ T cells compared with those in CHB liver tissues.^128^ It has shown that serum CTLA‐4 levels progressively increase from HBV carrier status through chronic hepatitis and cirrhosis to HCC, compared with healthy controls. Furthermore, CD8^+^ T cells in HCC tissues not only exhibit increased markers of exhaustion, including CTLA‐4, but also show a reduced production of effector cytokines such as IFN‐γ and TNF‐α, in contrast to CHB tissues. Additionally, the blockade of CTLA‐4 can enhance the anti‐HBV response and increase the expansion of HBV‐specific CD8^+^ T cells capable of producing IFN‐γ, uncovering its potential as a therapeutic target in HCC.^204^

In this review, we focus on the effects of PD‐1 and CTLA‐4 for TME and CD8^+^ T cells in HBV^+^HCC. Indeed, apart from PD‐1 and CTLA‐4, the complex regulation of immune checkpoints such as T‐cell immunoglobulin and ITIM domain, LAG‐3 and TIM‐3 in TME contributes to the main cause of immunosuppression.^205^ ICIs are being used clinically and have led to benefits for patients with HCC,^118^ a section that we will explain in more detail below.

### 4.6 Gut microbiota tunes CD8^+^ T cell immunity in HCC via the gut–liver axis

The gut–liver axis has been acknowledged to be a bidirectional communication between the liver and gut microbiota, with the latter playing a critical role in liver disease development, particularly in HCC, through metabolites and products of gut microbiota.^206^ Mechanisms by which the gut–liver axis contributes to the development of HCC in mouse models and patients, involving dysbiosis, bacterial metabolites and leaky gut. Among these, dysbiosis and leaky gut are prominent features of all stages of chronic liver disease, which facilitate the step‐by‐step progression of liver fibrosis, cirrhosis and HCC.^103^ Dysbiosis releases pro‐carcinogenic and pro‐aging metabolites, such as deoxycholic acid (DCA), and increases hepatic exposure to gut‐derived microbe‐associated molecular patterns, like LPS. Leaky gut, on the other hand, can promote chronic hepatic inflammation via Toll‐like receptor‐mediated signalling.^207^ Notably, the interactions between the gut microbiota and HCC are complex and multi‐faceted, and further studies are needed to gain insight into their relationship.

Gut microbiota composition differs significantly between different populations, specifically, a higher abundance of potentially anti‐inflammatory bacteria exists in HBV^+^HCC patients, comparing with healthy populations and HBV^−^/HCV^−^ HCC patients. Whereas the latter two populations have more potentially pro‐inflammatory bacteria in gut.^208^ Gut microbial diversity has been observed to decrease from healthy individuals to those with HBV^+^ cirrhosis but increase from HBV^+^ cirrhosis to early HBV^+^HCC. Concurrently, the abundance of butyrate‐producing bacteria, protective of intestinal mucosa and barrier function, is reduced, whereas that of LPS‐producing bacteria were increased, in early HCC compared with healthy controls.^209^ It identified specific microbial bacterial species (e.g. *Clostridium XIVa*, *Bacteroides*, *Lachnospiracea incertae sedis*) in patients with HBV^+^HCC, and suggested that serum bile acids may be a key mediator linking these microbial changes to the host transcriptome and potentially predicting clinical outcomes.^210^ However, the gut microbiota profiles in HCC patients can vary and sometimes contradict across studies, likely due to differences in patient characteristics, underlying liver disease aetiology, geographic location, race, diet and medications.^211^, ^212^

Moreover, imbalanced gut microbiota can directly influence the hepatic inflammatory microenvironment, promoting HCC development and progression. This imbalance may lead to the expansion of MDSCs and suppression of CD8^+^ T cells, with antibiotic treatment potentially interrupting this suppression.^110^ Yan's study found that compared with patients with HBV^+^ cirrhosis, patients with HBV^+^HCC had a significantly lower proportion of CD3^+^ and CD8^+^ T cells and an increased proportion of Tregs in their peripheral blood, which means that HBV^+^HCC exhibits a stronger immune‐suppressive response. Further, they found that this is closely related to the gut microbiota of HBV^+^HCC patients, such that in HBV^+^HCC patients, *Campilobacterota*, *Akkermaniacaeae* and CD8^+^ T cells were positively correlated at the phylum level; and *Prevotelaceae* and CD8^+^ T cells were significantly and positively correlated at the family level.^105^ A recent study by Ma et al.^213^ found that Bacteroides is the most significantly different genus and *B. thetaiotaomicron* to be the most significantly different species in Bacteroides when comparing faecal samples from patients in the HCC recurrent and HCC non‐recurrent groups. Further correlation analyses suggest that *B. thetaiotaomicron* may secrete acetic acid, contributing further to M1 macrophage polarisation and cytotoxic CD8^+^ T cell function, which ultimately suppresses the progression of tumours in HCC patients.^213^ Similarly, a significant correlation was observed between CD8^+^ T cell infiltration in melanoma tumours and specific gut microbiota, suggesting that the gut microbiota can modulate anti‐tumour immune responses.^214^

Additionally, growing evidence suggests that gut microbiota and their multiple metabolites are not only associated with the pathogenesis of HCC, but also with the treatment of HCC, especially ICIs.^215^, ^216^ 2,5­Dimethylcelecoxib (a derivative of celecoxib) may improve the TME of HCC by modulating gastrointestinal microbiota enrichment (*Bacteroides acidifaciens*, *Odoribacter laneus* and *Odoribacter splanchnicus*) in order to activate the AMPK–mTOR signalling pathway in CD4^+^ T cells, CD8^+^ T cells and NK cells, thereby enhancing IFN‐γ secretion and inhibit PD‐1 expression.^217^ The gut microbial metabolite butyrate can promote anti‐tumour therapeutic efficacy through inhibitor of DNA binding 2‐dependent modulation of CD8^+^ T cell immunity, suggesting that gut microbial metabolites may be effective as part of cancer therapy.^218^

In conclusion, the gut microbiota and its multiple metabolites not only profoundly influence the development of HCC, shaping the TME and affecting the function of CD8^+^ T cells, but also have a profound impact on the treatment of HCC.

### 4.7 CD8^+^ T cell‐based immunotherapy for HCC

#### 4.7.1 Immune checkpoint immunotherapy

ICIs are antibodies designed for preventing T‐cell exhaustion through inhibiting the interaction between immune checkpoint proteins and their ligands. These ICIs have emerged as the first immunotherapeutic medications proven effective in HCC.^219^ Approved ICIs, including Atezolizumab, Nivolumab and Pembrolizumab, have significantly transformed the landscape of HCC treatment.^220^ For instance, Nivolumab, a human anti‐PD‐1 monoclonal antibody, reactivates the anti‐tumour activity of suppressed effector T‐cells. It has shown promising efficacy in patients with advanced HCC, including those with chronic viral hepatitis, as demonstrated in a phase 1/2 dose‐escalation and expansion trial.^221^ Tremelimumab, another human monoclonal antibody targeting CTLA‐4, increases T‐cell activation and multiplication.^222^, ^223^

However, clinical trials have indicated that mono‐agent ICIs yield objective responses in only about 15% of patients with advanced HCC. Consequently, combinations of immunotherapies are being explored to increase T‐cell activation and expand the patient response range.^224^ For example, a phase I/II study showed that combining Tremelimumab (anti‐CTLA‐4 antibody) with Durvalumab (anti‐PD‐L1 antibody) offered better safety and more durable responses in HCC patients compared with either drug alone.^225^ Similarly, the combination of the anti‐PD‐1 antibody Sintilimab with Bevacizumab biosimilar (IBI305) improved overall survival (OS) in Chinese patients on late‐stage HBV^+^HCC.^226^

The pairing of the anti‐PD‐L1 antibody Atezolizumab with the anti‐VEGF‐A antibody Bevacizumab has set a new benchmark for first‐line treatment, achieving a median OS duration of 19 months in patients with advanced HCC.^119^ Additionally, the combination of IFN‐α and anti‐PD‐1 therapy has been identified as an efficient new strategy. IFN‐α modulates glucose metabolism in the HCC TME, enhancing the immune response induction by PD‐1 blockade.^227^ Furthermore, TOX, a critical regulator in T cell development and differentiation, has been identified as a potential target. Down‐regulation of TOX expression enhances the function of CD8^+^ T cells against tumours and displays a promising synergistic effect when used in combination with anti‐PD‐1 therapy.^200^ In conclusion, ICIs represent a significant advancement in therapy for advanced HCC. Ongoing research and clinical trials are expected to develop more types of ICIs and explore various combination therapies to further improve patient outcomes.

#### 4.7.2 Gene‐modified T cell therapy

Genetically modified T‐cell therapy, involving the use of T‐cells engineered to express tumour‐specific TCRs, is a promising approach in cancer treatment. There are two primary types of gene‐modified T‐cell therapies: TCR‐T therapy and CAR‐T therapy.^228^

TCR‐T therapy employs genetically modified T cells that express both the α and β chains of a tumour antigen‐specific TCR. The source of the TCR can be from a human sharing at least one specific HLA allele with the patient or from a genetically modified mouse with the same HLA allele immunised against a tumour antigen.^229^ This therapy is contingent upon the expression of major histocompatibility complex molecules and the target antigenic epitopes by tumour cells.^228^ In the context of HBV^+^HCC, several TCR‐T cell therapies are under development. In vitro and murine studies have shown that HBsAg‐TCR‐T cells are effective and safe for treating HBV^+^HCC.^230^ Clinical trials with autologous HBV‐TCR‐T cells have indicated feasibility, safety, tolerable toxicity and promising efficacy in advanced HBV^+^HCC patients.^231^ Enhancements in TCR‐T therapy may focus on extending T‐cell viability in vivo, preventing T‐cell exhaustion and improving tumour infiltration.^232^

CAR‐T therapy, another form of cellular immunotherapy, uses T cells engineered to express CARs.^233^, ^234^ CARs consist of an intracellular signalling domain, an intracellular antigen recognition domain and a membrane‐spanning structural domain. The intracellular domain is crucial for signalling and determining the immune response's efficacy, while the extracellular domain targets specific antigens.^235^ Common CAR‐T therapy targets include glypican‐3 (GPC3), epithelial cell adhesion molecule and mucin 1.^236^ GPC3, a carcinoembryonic antigen, overexpressed in HCC and involved in Wnt‐dependent cell proliferation, is a target for CAR (hYP7)‐T cells, which can eliminate GPC3‐positive HCC cells through perforin‐ and granzyme‐mediated apoptosis or Wnt signalling reduction in cancer cells.^237^, ^238^ Clinical trial of HCC already confirmed the safety and efficacy of CAR‐GPC3 T‐cell therapy.^238^, ^239^ Additionally, NKG2D ligand (NKG2DL) is another potential CAR‐T target, as it is typically absent in normal cells but overexpressed in malignant cells. It demonstrated that NKG2D‐BBz CAR‐T cells could specifically eradicate HCC cells in an NKG2DL‐dependent manner.^240^

However, the use of CAR‐T cells in solid malignancies, including HCC, faces challenges like susceptibility to the immune‐suppressive TME, decreased durability and proliferation of CAR‐T cells and limited traceability. Despite these challenges, genetically modified T‐cell therapies, particularly TCR‐T and CAR‐T, are evolving as effective treatments of late‐stage HCC, with ongoing research focusing on enhancing their effectiveness and overcoming existing limitations.^241^

#### 4.7.3 Cancer vaccine therapy

Cancer vaccines represent a form of immunotherapy that introduces tumour antigens into the patient's body in various forms, aiming to counteract the immunosuppressive TME and activate the immune system to combat cancer.^242^ For a cancer vaccine to be effective, it should ideally deliver the concentrated antigen to both HLA class I and class II molecules in DCs, thereby facilitating responses from both CD4^+^ and CD8^+^ T cells.^243^, ^244^ In the context of HCC, several specific cancer vaccines are under development. Alpha‐fetoprotein (AFP) vaccines, including DC vaccines, DNA vaccines and peptide vaccines, introduce AFP epitope peptides to APCs, leading to the generation of multiple AFP‐specific CTLs and induction of tumour immunity.^245^ The GPC3 peptide vaccine can induce peptide‐specific CTLs in HCC patients with positive GPC3 expression. This vaccine has the potential to transform ‘cold tumours’ into ‘hot tumours’, improving long‐term prognosis.^246^

However, monotherapy utilising cancer vaccines encounters substantial limitations in the prospective future for HCC. This is primarily due to the prevalent depression and impairment of immune functions in patients, rendering immune cells ineffectual in efficiently eliminating cancer cells. The integration of vaccine therapy with immune checkpoint blockades may enhance effectiveness and improve patient outcomes.^247^ For instance, consisting of seven highly immunogenic neoantigen peptides and clinical grade Poly(I:C), Neoantigen peptide vaccine (NeoVAC) for murine HCC cell line Hepa1­6 produces a powerful anti‐tumour immune response in a mouse model of HCC. The percentage of CD8^+^ Tissue‐resident memory T cells was significantly higher in the NeoVAC combined with PD‐1 blockade treatment group and positively correlated with anti‐tumour efficacy.^248^ Nevertheless, the current anti‐tumour efficacy of HCC cancer vaccines remains markedly limited and transient, underscoring the imperative for further refinement and development of more potent vaccine‐combined strategies.^249^

#### 4.7.4 Modulations of gut microbiota benefits CD8+ T cell‐based immunotherapy

The gut microbiota, due to the anatomical and functional links between the gut and liver, plays a significant role in the progression of HCC.^103^ Therapeutic modulation of the gut microbiota using antibiotics, probiotics, prebiotics and FMT emerges as a novel strategy for preventing the development of chronic hepatitis into HCC and for treating HCC,^250^ providing valuable supplementation for CD8^+^ T cell‐based immunotherapy.

Antibiotics target the gut–microbiota–liver axis and could potentially suppress HCC occurrence and progression. They reduce the total gut bacterial load and bacterial transport, thereby suppressing anti‐inflammatory signalling associated with leaky gut. Moreover, selective antibiotics can decrease the synthesis from microbial metabolites (e.g. DCA), which promotes HCC development.^207^ For example, oral antibiotic cocktail therapy (4Abx) in obesity‐induced HCC significantly reduced commensal gut microbiota, inhibiting hepatocyte senescence and HCC development.^251^ However, the simultaneous use of antibiotics during immunotherapy has been associated with higher mortality in advanced HCC patients.^252^ This suggests that our current understanding of gut microbiota is one‐sided, necessitating a more comprehensive exploration of antibiotic usage in this context.

Probiotics, beneficial microorganisms that inhabit the human gut, maintain gut microbiota balance, improve gut barrier function and potentially inhibit HCC development by altering gut microbiota composition.^253^ For instance, the probiotic formulation VSL#3 (containing eight different bacteria: one *Streptococcus thermophilus salivarius subgenus*, three *Bifidobacterium bifidums* and four *Lactobacillus lactis*) mitigated intestinal dysbiosis and inflammation and inhibited the progression of HCC in a DEN‐induced mouse model.^254^ Meanwhile, prebiotics, non‐absorbable oligosaccharides, promote the growth of beneficial bacteria, regulate immune responses and produce SCFA to inhibit HCC development.^206^ Resveratrol, for example, inhibited tumour growth in a subcutaneous Hepa1‐6 model of HCC and also increased the percentage of IFN‐γ‐expressing CD8^+^ T cells in both tumours and peripheral lymphoid organs.^255^

FMT, involving the infusion of faeces from a healthy donor into the gastrointestinal tract of a recipient,^256^, ^257^ has shown effectiveness for treatment *Clostridium difficile* infections^258^ and some chronic liver diseases like NAFLD.^250^ While its application in HCC treatment is still in the preclinical stage and requires further clinical studies,^253^, ^259^ FMT holds promise as a prospective direction, particularly when combined with existing therapeutic strategies. This is due to the pivotal role that gut microbiota plays in regulating immune responses. Notably, the groundbreaking report on FMT reversing the resistance of PD‐1 antibodies^260^ highlights the application value of FMT in HCC immunotherapy. However, FMT may be a double‐edged sword, meaning that it has great potential for treating diseases associated with alterations in the gut microbiota, but there are also concerns regarding the efficacy, safety and adverse effects of FMT treatments.^261^ A systematic review showed FMT‐related adverse incidents happened with 19% on FMT treatments. Of these, serious FMT‐related adverse events, including infections and death, were identified in 1.4% of cases.^262^ For the proper application of FMT therapy and to enhance therapeutic effectiveness and safety, the potentially pathological and poisonous properties of FMT preparations need to be assessed in vitro. Furthermore, the steady state of the patient's gut microbiota should be systematically tested and analysed before receiving FMT and the gut microbiota should be systematically compared and matched between recipients and donors.^263^

The modulations of the gut microbiota present a promising addition to CD8^+^ T cell‐based immunotherapy of HCC. However, the optimal combination strategy and approaches for utilising the modulations (e.g. FMT) alongside CD8^+^ T cells are yet to be fully established. Furthermore, this integration has not yet been widely implemented in clinical practice, necessitating further research.

Taken together, uncontrolled chronic HBV infection may gradually develop into fatal HCC. The TME of HBV^+^HCC developed in this process is remarkably complicated. As the main anti‐tumour cells of TME, the quantity and function of CD8^+^ T cells decide the anti‐tumour efficacy as well as influence the diagnosis to a great extent. However, CD8^+^ T cells are often exposed to various challenges in TME, such as hypoxia, lactate, metabolism reprogramming of amino acid and up‐regulation of immune checkpoints (e.g. PD‐1, CTLA‐4). In immunosuppressive TME, CD8^+^ T cells are progressively exhausted, showing reduced proliferative capacity and effector function. Apart from CD8^+^ T cells, other kinds of immune cells are present in the TME of HCC, such as MDCSs, DCs and Tregs, which also have an impact on the immune function of CD8^+^ T cells. Additionally, gut microbiota imbalance directly or indirectly shapes the TME through the gut–liver axis, with consequent effects on CD8^+^ T cells. At present, there are a number of anti‐tumour therapeutic strategies targeting TME for HCC, such as ICIs, genetically modified T‐cell therapies and gut microbiota modulation, which have benefited numerous HCC patients (Table 2). However, they still have some shortcomings, such as the immunological resistance issues that remain a key limitation for the growth of ICIs.^205^ Therefore, the effect of TME on CD8^+^ T cells in HBV^+^HCC requires further in‐depth studies, as well as the mechanisms of various HCC therapeutic strategies need to be explored and validated by further research and clinical trials.

**TABLE 2:** Therapeutic interventions for HCC based on CD8^+^ T cells.

Therapeutic intervention	Mechanism of action and efficacy	Specific treatment drugs and methods	Clinical trial number and references
Immune checkpoint immunotherapy	Preventing T‐cell exhaustion by inhibiting the interaction between immune checkpoint proteins and their ligands.	Atezolizumab	NCT02715531^264^
		Nivolumab	NCT01658878^221^
		Pembrolizumab	NCT02702414^265^
		Tremelimumab	NCT01008358^266^
		Tremelimumab + Durvalumab	NCT02519348^225^
		Sintilimab + Bevacizumab biosimilar (IBI305)	NCT03794440^226^
		Atezolizumab + Bevacizumab	NCT02715531^264^
		Anti–PD‐1 antibody + IFN‐α	^227^
T cell receptor engineered T‐cell	T‐cells modified to express tumour‐specific TCRs.	HBsAg­specific Ai­TCR‐T cells	^230^
		HBV‐TCR‐T‐cells	NCT03899415^231^
Chimeric antigen receptor engineered T‐cell	T‐cells engineered to express CARs which consist of an intracellular signalling domain, an extracellular antigen recognition domain and a transmembrane domain.	CAR (hYP7) T cells	^238^
		GPC3 CAR‐T cells	NCT02395250^239^
		NKG2D‐BBz CAR‐T cells	^240^
Cancer vaccine therapy	Introducing tumour antigens into the patient's body in various forms, aiming to counteract the immunosuppressive TME and activate the immune system to combat cancer.	GPC3 vaccine	UMIN000002614^246^
		AFP vaccine + Anti‐PD‐L1 antibody	^247^
		NeoVAC + Anti‐PD‐1 antibody	^248^
Antibiotics	Modulations of gut microbiota: Reducing the total gut bacterial load and bacterial translocation, thereby inhibiting pro‐inflammatory signals associated with leaky gut.	A combination of four antibiotics (4Abx) of ampicillin, neomycin, metronidazole and vancomycin	^251^
Probiotics	Modulations of gut microbiota: Maintaining gut microbiota balance, improve gut barrier function and potentially inhibit HCC development by altering gut microbiota composition.	VSL#3 (containing eight different bacteria: one *Streptococcus thermophilus salivarius subgenus*, three *Bifidobacterium bifidums* and four *Lactobacillus lactis*)	^254^
Prebiotics	Modulations of gut microbiota: Promoting the growth of beneficial bacteria, regulate immune responses and produce short‐chain fatty acids to inhibit HCC development.	Resveratrol	^255^
Faecal microbiota transplantation	Modulations of gut microbiota: Infusion of faeces from a healthy donor into the recipient's gastrointestinal tract to treat diseases associated with alterations in the gut microbiota.	–	^258^, ^267^, ^268^

Abbreviations: AFP, alpha‐fetoprotein.; Ai, affinity‐improved; CARs, chimeric antigen receptors; CAR‐T cells, chimeric antigen receptor engineered T‐cells; GPC3, glypican‐3; HBsAg, hepatitis B surface antigen; HCC, hepatocellular carcinoma; hYP7, humanised YP7; IFN‐α, interferon α; NeoVAC, neoantigen peptide vaccine; NKG2D, natural killer group 2 member D; PD‐1, programmed cell death protein‐1; PD‐L1, programmed death ligand‐1; TCRs, T cell receptors; TCR‐T cells, T cell receptor engineered T‐cells; TME, tumour microenvironment.

## 5 CONCLUSION

In this review, we have comprehensively summarised the impact of microenvironmental changes, such as hypoxia, acidosis and metabolic reprogramming, on CD8^+^ T cells during the progression from HBV infection to HCC (Figure 2). The TME of HCC, comprising tumour cells, a variety of immune cells (e.g. CD8^+^ T cells) and stromal cells, exhibits significant heterogeneity, which is a major contributor to HCC metastasis, relapse and drug resistance.^269^ Increasing attention is being paid to the TME in HCC, with research methods like single‐cell analysis and cytometry by time‐of‐flight being utilised.^270^, ^271^, ^272^ The microenvironment in HBV^+^HCC development is notably immunosuppressive to CD8^+^ T cells, leading to higher expression of exhaustion markers, reduced proliferation and cellular activity and diminished effector cytokine production, all contributing to HCC progression. Additionally, the gut microbiota indirectly influences the immune microenvironment, thereby affecting the function and phenotype of CD8^+^ T cells.^218^, ^273^ However, the interplay of various factors shaping the immune microenvironment is complex, and current studies often focus on a single or limited number of factors. The molecular mechanisms underlying HBV‐induced HCC and its impact on CD8^+^ T cells require further exploration. Advanced methods to mimic the complex in vivo immune microenvironment, such as organoids, may provide deeper insights into CD8^+^ T cell roles and changes in HBV^+^HCC.^274^

Since the immune system plays a critical role in controlling cancer progression, the exhaustion of CD8^+^ T cells can lead to ineffective HBV clearance, influencing the progression of HBV infection‐related diseases.^116^, ^219^ Current research in treating HBV‐related diseases focuses on immunotherapies like ICIs, cancer vaccines and gene‐modified T cell therapy (Figure 4). However, single‐agent immunotherapies have limitations, such as limited efficacy and drug resistance. For instance, single‐agent ICIs yield objective remission rates of only 15−20% in patients with advanced HCC and often lack significant OS benefits, with intrinsic resistance observed in about 30% of HCC cases.^224^ Thus, developing combination therapies and novel immunotherapeutic agents is a research priority.

Targeting the gut microbiota has become one of the new strategies of preventing and treating HBV^+^HCC. Liver organoids, an innovative approach, offer valuable models for exploring liver‐related disease mechanisms and potential applications in therapy, liver regeneration and drug screening.^274^, ^275^, ^276^ The future of HBV^+^HCC treatment is likely to see the approval of more therapeutics and the necessity for personalised treatment regimens to maximise clinical benefits for patients.

## AUTHOR CONTRIBUTIONS

Yangzhe Wu, Bing Yue and Yuxia Gao concepted, drafted and revised the review and prepared figures. Yi Hu critically reviewed and revised the article. Ligong Lu and Meixiao Zhan provided critical suggestions and resources. All authors have read and approved the article.

## CONFLICT OF INTEREST STATEMENT

All authors declare no conflict of interest on this work.

## ETHICS STATEMENT

Not applicable.

## References

[1] Sung H , Ferlay J , Siegel RL , et al. Global cancer statistics 2020: GLOBOCAN estimates of incidence and mortality worldwide for 36 cancers in 185 countries. CA Cancer J Clin. 2021;71:209‐249. 33538338 10.3322/caac.21660

[2] Rumgay H , Arnold M , Ferlay J , et al. Global burden of primary liver cancer in 2020 and predictions to 2040. J Hepatol. 2022;77:1598‐1606. 36208844 10.1016/j.jhep.2022.08.021 PMC9670241

[3] Vogel A , Meyer T , Sapisochin G , Salem R , Saborowski A . Hepatocellular carcinoma. Lancet. 2022;400:1345‐1362. 36084663 10.1016/S0140-6736(22)01200-4

[4] McGlynn KA , Petrick JL , El‐Serag HB . Epidemiology of hepatocellular carcinoma. Hepatology. 2021;73(1). Suppl. 10.1002/hep.31288 PMC7577946 32319693

[5] Kubes P , Jenne C . Immune responses in the liver. Annu Rev Immunol. 2018;36:247‐277. 29328785 10.1146/annurev-immunol-051116-052415

[6] Robinson MW , Harmon C , O'Farrelly C . Liver immunology and its role in inflammation and homeostasis. Cell Mol Immunol. 2016;13:267‐276. 27063467 10.1038/cmi.2016.3 PMC4856809

[7] Zheng J‐R , Wang Z‐L , Feng B . Hepatitis B functional cure and immune response. Front Immunol. 2022;13:1075916. 36466821 10.3389/fimmu.2022.1075916 PMC9714500

[8] Iannacone M , Guidotti LG . Immunobiology and pathogenesis of hepatitis B virus infection. Nat Rev Immunol. 2022;22:19‐32. 34002067 10.1038/s41577-021-00549-4

[9] Zhao H‐J , Hu Y‐F , Han Q‐J , Zhang J . Innate and adaptive immune escape mechanisms of hepatitis B virus. World J Gastroenterol. 2022;28:881‐896. 35317051 10.3748/wjg.v28.i9.881 PMC8908287

[10] Han JW , Shin E‐C . Liver‐resident memory CD8+ T cells: possible roles in chronic HBV infection. Int J Mol Sci. 2020;22(1):283. 33396596 10.3390/ijms22010283 PMC7795050

[11] Bertoletti A , Ferrari C . Adaptive immunity in HBV infection. J Hepatol. 2016;64:S71‐S83. 27084039 10.1016/j.jhep.2016.01.026

[12] Luxenburger H , Neumann‐Haefelin C . Liver‐resident CD8+ T cells in viral hepatitis: not always good guys. J Clin Invest. 2023;133(1):e165033. 36594469 10.1172/JCI165033 PMC9797333

[13] Wilson GK , Tennant DA , McKeating JA . Hypoxia inducible factors in liver disease and hepatocellular carcinoma: current understanding and future directions. J Hepatol. 2014;61:1397‐1406. 25157983 10.1016/j.jhep.2014.08.025

[14] Yoo Y‐G , Oh SH , Park ES , et al. Hepatitis B virus X protein enhances transcriptional activity of hypoxia‐inducible factor‐1alpha through activation of mitogen‐activated protein kinase pathway. J Biol Chem. 2003;278:39076‐39084. 12855680 10.1074/jbc.M305101200

[15] Hu J‐L , Liu LP , Yang SL , et al. Hepatitis B virus induces hypoxia‐inducible factor‐2α expression through hepatitis B virus X protein. Oncol Rep. 2016;35:1443‐1448. 26647960 10.3892/or.2015.4480

[16] Choudhry H , Harris AL . Advances in hypoxia‐inducible factor biology. Cell Metab. 2018;27:281‐298. 29129785 10.1016/j.cmet.2017.10.005

[17] Kim C‐H . Hidden secret in hepatitis B viral X protein mutation and hypoxia‐inducible factor‐1α in hepatocarcinoma cancer. Hepatobiliary Surg Nutr. 2014;3:115‐117. 25019072 10.3978/j.issn.2304-3881.2014.02.14 PMC4073312

[18] Wing PAC , Liu PJ , Harris JM , et al. Hypoxia inducible factors regulate hepatitis B virus replication by activating the basal core promoter. J Hepatol. 2021;75:64‐73. 33516779 10.1016/j.jhep.2020.12.034 PMC8214165

[19] Huang H , Yuan D , Li M , et al. Active HBV replication in hypoxic pericentral zone 3 is upregulated by multiple host factors including HIF‐1α. J Hepatol. 2022;77:265‐267. 35219790 10.1016/j.jhep.2022.01.031

[20] Lee P , Chandel NS , Simon MC . Cellular adaptation to hypoxia through hypoxia inducible factors and beyond. Nat Rev Mol Cell Biol. 2020;21:268‐283. 32144406 10.1038/s41580-020-0227-y PMC7222024

[21] Barili V , Boni C , Rossi M , et al. Metabolic regulation of the HBV‐specific T cell function. Antiviral Res. 2021;185:104989. 33248194 10.1016/j.antiviral.2020.104989

[22] Schurich A , Pallett LJ , Jajbhay D , et al. Distinct metabolic requirements of exhausted and functional virus‐specific CD8 T cells in the same host. Cell Rep. 2016;16:1243‐1252. 27452473 10.1016/j.celrep.2016.06.078 PMC4977274

[23] Cretenet G , Clerc I , Matias M , et al. Cell surface Glut1 levels distinguish human CD4 and CD8 T lymphocyte subsets with distinct effector functions. Sci Rep. 2016;6:24129. 27067254 10.1038/srep24129 PMC4828702

[24] Taylor CT , Scholz CC . The effect of HIF on metabolism and immunity. Nat Rev Nephrol. 2022;18:573‐587. 35726016 10.1038/s41581-022-00587-8 PMC9208707

[25] Phan AT , Goldrath AW . Hypoxia‐inducible factors regulate T cell metabolism and function. Mol Immunol. 2015;68:527‐535. 26298577 10.1016/j.molimm.2015.08.004 PMC4679538

[26] Xu Y , Chaudhury A , Zhang M , et al. Glycolysis determines dichotomous regulation of T cell subsets in hypoxia. J Clin Invest. 2016;126:2678‐2688. 27294526 10.1172/JCI85834 PMC4922684

[27] Matsufuji S , Kitajima Y , Higure K , et al. A HIF‐1α inhibitor combined with palmitic acid and L‐carnitine treatment can prevent the fat metabolic reprogramming under hypoxia and induce apoptosis in hepatocellular carcinoma cells. Cancer Metab. 2023;11:25. 38066600 10.1186/s40170-023-00328-w PMC10709876

[28] Yoo HC , Park SJ , Nam M , et al. A variant of SLC1A5 is a mitochondrial glutamine transporter for metabolic reprogramming in cancer cells. Cell Metab. 2020;31(2):267‐283.e12. 31866442 10.1016/j.cmet.2019.11.020

[29] Mittal M , Siddiqui MR , Tran K , Reddy SP , Malik AB . Reactive oxygen species in inflammation and tissue injury. Antioxid Redox Signal. 2014;20:1126‐1167. 23991888 10.1089/ars.2012.5149 PMC3929010

[30] Zhang B , Liu SQ , Li C , et al. MicroRNA‐23a curbs necrosis during early T cell activation by enforcing intracellular reactive oxygen species equilibrium. Immunity. 2016;44:568‐581. 26921109 10.1016/j.immuni.2016.01.007 PMC4794397

[31] Schieber M , Chandel NS . ROS function in redox signaling and oxidative stress. Curr Biol. 2014;24:R453‐R462. 24845678 10.1016/j.cub.2014.03.034 PMC4055301

[32] Fisicaro P , et al. Proteasome dysfunction as a reversible defect underlying virus‐specific CD8 cell exhaustion in chronic hepatitis B. J Hepatol. 2017;66:S30‐S30.

[33] Acerbi G , Montali I , Ferrigno GD , et al. Functional reconstitution of HBV‐specific CD8 T cells by in vitro polyphenol treatment in chronic hepatitis B. J Hepatol. 2021;74:783‐793. 33188902 10.1016/j.jhep.2020.10.034

[34] Korovila I , Hugo M , Castro JP , et al. Proteostasis, oxidative stress and aging. Redox Biol. 2017;13:550‐567. 28763764 10.1016/j.redox.2017.07.008 PMC5536880

[35] Gumeni S , Trougakos IP . Cross talk of proteostasis and mitostasis in cellular homeodynamics, ageing, and disease. Oxid Med Cell Longev. 2016;2016:4587691. 26977249 10.1155/2016/4587691 PMC4763003

[36] Zhang C , Li J , Cheng Y , et al. Single‐cell RNA sequencing reveals intrahepatic and peripheral immune characteristics related to disease phases in HBV‐infected patients. Gut. 2023;72:153‐167. 35361683 10.1136/gutjnl-2021-325915 PMC9763233

[37] Ye B , Liu X , Li X , Kong H , Tian L , Chen Y . T‐cell exhaustion in chronic hepatitis B infection: current knowledge and clinical significance. Cell Death Dis. 2015;6:e1694. 25789969 10.1038/cddis.2015.42 PMC4385920

[38] Wherry EJ . T cell exhaustion. Nat Immunol. 2011;12:492‐499. 21739672 10.1038/ni.2035

[39] Noman MZ , Desantis G , Janji B , et al. PD‐L1 is a novel direct target of HIF‐1α, and its blockade under hypoxia enhanced MDSC‐mediated T cell activation. J Exp Med. 2014;211:781‐790. 24778419 10.1084/jem.20131916 PMC4010891

[40] Cubillos‐Zapata C , Avendaño‐Ortiz J , Hernandez‐Jimenez E , et al. Hypoxia‐induced PD‐L1/PD‐1 crosstalk impairs T‐cell function in sleep apnoea. Eur Respir J. 2017;50(4):1700833. 29051270 10.1183/13993003.00833-2017

[41] Bengsch B , Johnson AL , Kurachi M , et al. Bioenergetic insufficiencies due to metabolic alterations regulated by the inhibitory receptor PD‐1 are an early driver of CD8(+) T cell exhaustion. Immunity. 2016;45:358‐373. 27496729 10.1016/j.immuni.2016.07.008 PMC4988919

[42] Ai L , Xu A , Xu J . Roles of PD‐1/PD‐L1 pathway: signaling, cancer, and beyond. Adv Exp Med Biol. 2020;1248:33‐59. 32185706 10.1007/978-981-15-3266-5_3

[43] Yokosuka T , Takamatsu M , Kobayashi‐Imanishi W , Hashimoto‐Tane A , Azuma M , Saito T . Programmed cell death 1 forms negative costimulatory microclusters that directly inhibit T cell receptor signaling by recruiting phosphatase SHP2. J Exp Med. 2012;209:1201‐1217. 22641383 10.1084/jem.20112741 PMC3371732

[44] Patsoukis N , Bardhan K , Chatterjee P , et al. PD‐1 alters T‐cell metabolic reprogramming by inhibiting glycolysis and promoting lipolysis and fatty acid oxidation. Nat Commun. 2015;6:6692. 25809635 10.1038/ncomms7692 PMC4389235

[45] Tian Y , Kuo C‐F , Akbari O , Ou J‐HJ . Maternal‐derived hepatitis B virus e antigen alters macrophage function in offspring to drive viral persistence after vertical transmission. Immunity. 2016;44:1204‐1214. 27156385 10.1016/j.immuni.2016.04.008 PMC4871724

[46] Lopes AR , Kellam P , Das A , et al. Bim‐mediated deletion of antigen‐specific CD8 T cells in patients unable to control HBV infection. J Clin Invest. 2008;118:1835‐1845. 18398508 10.1172/JCI33402 PMC2289792

[47] Horst AK , Neumann K , Diehl L , Tiegs G . Modulation of liver tolerance by conventional and nonconventional antigen‐presenting cells and regulatory immune cells. Cell Mol Immunol. 2016;13:277‐292. 27041638 10.1038/cmi.2015.112 PMC4856800

[48] Boni C , Lampertico P , Talamona L , et al. Natural killer cell phenotype modulation and natural killer/T‐cell interplay in nucleos(t)ide analogue‐treated hepatitis e antigen‐negative patients with chronic hepatitis B. Hepatology. 2015;62:1697‐1709. 26361374 10.1002/hep.28155

[49] Yu J , Green MD , Li S , et al. Liver metastasis restrains immunotherapy efficacy via macrophage‐mediated T cell elimination. Nat Med. 2021;27:152‐164. 33398162 10.1038/s41591-020-1131-x PMC8095049

[50] Lercher A , Popa AM , Viczenczova C , et al. Hepatocyte‐intrinsic type I interferon signaling reprograms metabolism and reveals a novel compensatory mechanism of the tryptophan‐kynurenine pathway in viral hepatitis. PLoS Pathog. 2020;16:e1008973. 33045014 10.1371/journal.ppat.1008973 PMC7580883

[51] Campesato LF , Budhu S , Tchaicha J , et al. Blockade of the AHR restricts a Treg‐macrophage suppressive axis induced by L‐Kynurenine. Nat Commun. 2020;11:4011. 32782249 10.1038/s41467-020-17750-z PMC7419300

[52] Nüse B , Holland T , Rauh M , Gerlach RG , Mattner J . L‐arginine metabolism as pivotal interface of mutual host‐microbe interactions in the gut. Gut Microbes. 2023;15:2222961. 37358082 10.1080/19490976.2023.2222961 PMC10294761

[53] Pallett LJ , Gill US , Quaglia A , et al. Metabolic regulation of hepatitis B immunopathology by myeloid‐derived suppressor cells. Nat Med. 2015;21:591‐600. 25962123 10.1038/nm.3856 PMC4458139

[54] Yang F , Yu X , Zhou C , et al. Hepatitis B e antigen induces the expansion of monocytic myeloid‐derived suppressor cells to dampen T‐cell function in chronic hepatitis B virus infection. PLoS Pathog. 2019;15:e1007690. 30998767 10.1371/journal.ppat.1007690 PMC6472891

[55] Fisicaro P , Barili V , Rossi M , et al. Pathogenetic mechanisms of T cell dysfunction in chronic HBV infection and related therapeutic approaches. Front Immunol. 2020;11:849. 32477347 10.3389/fimmu.2020.00849 PMC7235343

[56] Mondanelli G , Bianchi R , Pallotta MT , et al. A relay pathway between arginine and tryptophan metabolism confers immunosuppressive properties on dendritic cells. Immunity. 2017;46:233‐244. 28214225 10.1016/j.immuni.2017.01.005 PMC5337620

[57] Peppa D , Gill US , Reynolds G , et al. Up‐regulation of a death receptor renders antiviral T cells susceptible to NK cell‐mediated deletion. J Exp Med. 2013;210(1):99‐114. 23254287 10.1084/jem.20121172 PMC3549717

[58] Li H , Zhai N , Wang Z , et al. Regulatory NK cells mediated between immunosuppressive monocytes and dysfunctional T cells in chronic HBV infection. Gut. 2018;67:2035‐2044. 28899983 10.1136/gutjnl-2017-314098 PMC6176520

[59] Crispe IN . Immune tolerance in liver disease. Hepatology. 2014;60:2109‐2117. 24913836 10.1002/hep.27254 PMC4274953

[60] Baudi I , Kawashima K , Isogawa M . HBV‐specific CD8+ T‐cell tolerance in the liver. Front Immunol. 2021;12:721975. 34421926 10.3389/fimmu.2021.721975 PMC8378532

[61] Alfei F , Kanev K , Hofmann M , et al. TOX reinforces the phenotype and longevity of exhausted T cells in chronic viral infection. Nature. 2019;571:265‐269. 31207605 10.1038/s41586-019-1326-9

[62] Bordon Y . TOX for tired T cells. Nat Rev Immunol. 2019;19:476. 31243349 10.1038/s41577-019-0193-9

[63] Khan O , Giles JR , McDonald S , et al. TOX transcriptionally and epigenetically programs CD8+ T cell exhaustion. Nature. 2019;571:211‐218. 31207603 10.1038/s41586-019-1325-x PMC6713202

[64] Sekine T , Perez‐Potti A , Nguyen S , et al. TOX is expressed by exhausted and polyfunctional human effector memory CD8+ T cells. Sci Immunol. 2020;5(49):eaba7918. 32620560 10.1126/sciimmunol.aba7918

[65] Yao C , Sun HW , Lacey NE , et al. Single‐cell RNA‐seq reveals TOX as a key regulator of CD8+ T cell persistence in chronic infection. Nat Immunol. 2019;20:890‐901. 31209400 10.1038/s41590-019-0403-4 PMC6588409

[66] Heim K , Binder B , Sagar , et al. TOX defines the degree of CD8+ T cell dysfunction in distinct phases of chronic HBV infection. Gut. 2020;70:1550‐1560. 33097558 10.1136/gutjnl-2020-322404 PMC8292571

[67] Zhou Y‐J , Li G , Wang J , et al. PD‐L1: expression regulation. Blood Sci. 2023;5:77‐91. 37228770 10.1097/BS9.0000000000000149 PMC10205351

[68] Yi M , Zheng X , Niu M , Zhu S , Ge H , Wu K . Combination strategies with PD‐1/PD‐L1 blockade: current advances and future directions. Mol Cancer. 2022;21:28. 35062949 10.1186/s12943-021-01489-2 PMC8780712

[69] Fisicaro P , Barili V , Montanini B , et al. Targeting mitochondrial dysfunction can restore antiviral activity of exhausted HBV‐specific CD8 T cells in chronic hepatitis B. Nat Med. 2017;23:327‐336. 28165481 10.1038/nm.4275

[70] Hackstein C‐P , Spitzer J , Symeonidis K , et al. Interferon‐induced IL‐10 drives systemic T‐cell dysfunction during chronic liver injury. J Hepatol. 2023;79:150‐166. 36870611 10.1016/j.jhep.2023.02.026

[71] Schurich A , Pallett LJ , Lubowiecki M , et al. The third signal cytokine IL‐12 rescues the anti‐viral function of exhausted HBV‐specific CD8 T cells. PLoS Pathog. 2013;9:e1003208. 23516358 10.1371/journal.ppat.1003208 PMC3597507

[72] Bénéchet AP , De Simone G , Di Lucia P , et al. Dynamics and genomic landscape of CD8+ T cells undergoing hepatic priming. Nature. 2019;574:200‐205. 31582858 10.1038/s41586-019-1620-6 PMC6858885

[73] Boni C , Barili V , Acerbi G , et al. HBV immune‐therapy: from molecular mechanisms to clinical applications. Int J Mol Sci. 2019;20(11):2754. 31195619 10.3390/ijms20112754 PMC6600394

[74] Boni C , Janssen HLA , Rossi M , et al. Combined GS‐4774 and tenofovir therapy can improve HBV‐specific T‐cell responses in patients with chronic hepatitis. Gastroenterology. 2019;157(1):227‐241.e7. 30930022 10.1053/j.gastro.2019.03.044

[75] Lok AS , Pan CQ , Han SH , et al. Randomized phase II study of GS‐4774 as a therapeutic vaccine in virally suppressed patients with chronic hepatitis B. J Hepatol. 2016;65:509‐516. 27210427 10.1016/j.jhep.2016.05.016

[76] Zhou C , Li C , Gong GZ , et al. Analysis of immunological mechanisms exerted by HBsAg‐HBIG therapeutic vaccine combined with Adefovir in chronic hepatitis B patients. Hum Vaccin Immunother. 2017;13:1989‐1996. 28665747 10.1080/21645515.2017.1335840 PMC5612521

[77] Kosinska AD , Bauer T , Protzer U . Therapeutic vaccination for chronic hepatitis B. Curr Opin Virol. 2017;23:75‐81. 28453967 10.1016/j.coviro.2017.03.011

[78] Bertoletti A , Tan AT . HBV as a target for CAR or TCR‐T cell therapy. Curr Opin Immunol. 2020;66:35‐41. 32361634 10.1016/j.coi.2020.04.003

[79] Krebs K , Böttinger N , Huang LR , et al. T cells expressing a chimeric antigen receptor that binds hepatitis B virus envelope proteins control virus replication in mice. Gastroenterology. 2013;145:456‐465. 23639914 10.1053/j.gastro.2013.04.047

[80] Qasim W , Brunetto M , Gehring AJ , et al. Immunotherapy of HCC metastases with autologous T cell receptor redirected T cells, targeting HBsAg in a liver transplant patient. J Hepatol. 2015;62:486‐491. 25308176 10.1016/j.jhep.2014.10.001

[81] Kruse RL , Shum T , Tashiro H , et al. HBsAg‐redirected T cells exhibit antiviral activity in HBV‐infected human liver chimeric mice. Cytotherapy. 2018;20:697‐705. 29631939 10.1016/j.jcyt.2018.02.002 PMC6038120

[82] Zoulim F , Fournier C , Habersetzer F , et al. Safety and immunogenicity of the therapeutic vaccine TG1050 in chronic hepatitis B patients: a phase 1b placebo‐controlled trial. Hum Vaccin Immunother. 2020;16:388‐399. 31373537 10.1080/21645515.2019.1651141 PMC7158919

[83] Mahtab Al , Akbar SMF , Aguilar JC , et al. Treatment of chronic hepatitis B naïve patients with a therapeutic vaccine containing HBs and HBc antigens (a randomized, open and treatment controlled phase III clinical trial). PLoS One. 2018;13:e0201236. 30133478 10.1371/journal.pone.0201236 PMC6104936

[84] Gaggar A , Coeshott C , Apelian D , et al. Safety, tolerability and immunogenicity of GS‐4774, a hepatitis B virus‐specific therapeutic vaccine, in healthy subjects: a randomized study. Vaccine. 2014;32:4925‐4931. 25045824 10.1016/j.vaccine.2014.07.027

[85] Koh S , Kah J , Tham CYL , et al. Nonlytic lymphocytes engineered to express virus‐specific T‐cell receptors limit HBV infection by activating APOBEC3. Gastroenterology. 2018;155(1):180‐193.e6. 29550589 10.1053/j.gastro.2018.03.027

[86] Ju C , Colgan SP , Eltzschig HK . Hypoxia‐inducible factors as molecular targets for liver diseases. J Mol Med (Berl). 2016;94:613‐627. 27094811 10.1007/s00109-016-1408-1 PMC4879168

[87] Affo S , Yu L‐X , Schwabe RF . The role of cancer‐associated fibroblasts and fibrosis in liver cancer. Annu Rev Pathol. 2017;12:153‐186. 27959632 10.1146/annurev-pathol-052016-100322 PMC5720358

[88] Wang S , Friedman SL . Hepatic fibrosis: a convergent response to liver injury that is reversible. J Hepatol. 2020;73:210‐211. 32402525 10.1016/j.jhep.2020.03.011 PMC10664493

[89] Tuchendler E , Tuchendler PK , Madej G . Immunodeficiency caused by cirrhosis. Clin Exp Hepatol. 2018;4:158‐164. 30324140 10.5114/ceh.2018.78119 PMC6185932

[90] Koda Y , Teratani T , Chu P‐S , et al. CD8+ tissue‐resident memory T cells promote liver fibrosis resolution by inducing apoptosis of hepatic stellate cells. Nat Commun. 2021;12:4474. 34294714 10.1038/s41467-021-24734-0 PMC8298513

[91] Muhanna N , Doron S , Wald O , et al. Activation of hepatic stellate cells after phagocytosis of lymphocytes: a novel pathway of fibrogenesis. Hepatology. 2008;48:963‐977. 18726940 10.1002/hep.22413 PMC2880478

[92] Liu Y , Dong Y , Wu X , et al. Identification of immune microenvironment changes and the expression of immune‐related genes in liver cirrhosis. Front Immunol. 2022;13:918445. 35903097 10.3389/fimmu.2022.918445 PMC9315064

[93] Cai J , Hu M , Chen Z , Ling Z . The roles and mechanisms of hypoxia in liver fibrosis. J Transl Med. 2021;19:186. 33933107 10.1186/s12967-021-02854-x PMC8088569

[94] Vuillefroy de Silly R , Ducimetière L , Yacoub Maroun C , et al. Phenotypic switch of CD8(+) T cells reactivated under hypoxia toward IL‐10 secreting, poorly proliferative effector cells. Eur J Immunol. 2015;45:2263‐2275. 25929785 10.1002/eji.201445284 PMC7163737

[95] Lebossé F , Gudd C , Tunc E , et al. CD8+T cells from patients with cirrhosis display a phenotype that may contribute to cirrhosis‐associated immune dysfunction. EBioMedicine. 2019;49:258‐268. 31678004 10.1016/j.ebiom.2019.10.011 PMC6945243

[96] Bernardi M , Moreau R , Angeli P , et al. Mechanisms of decompensation and organ failure in cirrhosis: from peripheral arterial vasodilation to systemic inflammation hypothesis. J Hepatol. 2015;63:1272‐1284. 26192220 10.1016/j.jhep.2015.07.004

[97] Moroni F , Dwyer BJ , Graham C , et al. Safety profile of autologous macrophage therapy for liver cirrhosis. Nat Med. 2019;25:1560‐1565. 31591593 10.1038/s41591-019-0599-8

[98] Pellicoro A , Ramachandran P , Iredale JP , Fallowfield JA . Liver fibrosis and repair: immune regulation of wound healing in a solid organ. Nat Rev Immunol. 2014;14:181‐194. 24566915 10.1038/nri3623

[99] Tripathi A , Debelius J , Brenner DA , et al. The gut‐liver axis and the intersection with the microbiome. Nat Rev Gastroenterol Hepatol. 2018;15:397‐411. 29748586 10.1038/s41575-018-0011-z PMC6319369

[100] Wang J , Wang Y , Zhang X , et al. Gut microbial dysbiosis is associated with altered hepatic functions and serum metabolites in chronic hepatitis B patients. Front Microbiol. 2017;8:2222. 29180991 10.3389/fmicb.2017.02222 PMC5693892

[101] Woodhouse CA , Patel VC , Singanayagam A , Shawcross DL . Review article: the gut microbiome as a therapeutic target in the pathogenesis and treatment of chronic liver disease. Aliment Pharmacol Ther. 2018;47:192‐202. 29083037 10.1111/apt.14397

[102] Caussy C , Tripathi A , Humphrey G , et al. A gut microbiome signature for cirrhosis due to nonalcoholic fatty liver disease. Nat Commun. 2019;10:1406. 30926798 10.1038/s41467-019-09455-9 PMC6440960

[103] Schwabe RF , Greten TF . Gut microbiome in HCC—Mechanisms, diagnosis and therapy. J Hepatol. 2020;72:230‐238. 31954488 10.1016/j.jhep.2019.08.016

[104] Aron‐Wisnewsky J , Vigliotti C , Witjes J , et al. Gut microbiota and human NAFLD: disentangling microbial signatures from metabolic disorders. Nat Rev Gastroenterol Hepatol. 2020;17:279‐297. 32152478 10.1038/s41575-020-0269-9

[105] Yan F , Zhang Q , Shi K , et al. Gut microbiota dysbiosis with hepatitis B virus liver disease and association with immune response. Front Cell Infect Microbiol. 2023;13:1152987. 37201112 10.3389/fcimb.2023.1152987 PMC10185817

[106] Bajaj JS , Heuman DM , Hylemon PB , et al. Altered profile of human gut microbiome is associated with cirrhosis and its complications. J Hepatol. 2014;60:940‐947. 24374295 10.1016/j.jhep.2013.12.019 PMC3995845

[107] Bajaj JS , Betrapally NS , Hylemon PB , et al. Salivary microbiota reflects changes in gut microbiota in cirrhosis with hepatic encephalopathy. Hepatology. 2015;62:1260‐1271. 25820757 10.1002/hep.27819 PMC4587995

[108] Solé C , Guilly S , Da Silva K , et al. Alterations in gut microbiome in cirrhosis as assessed by quantitative metagenomics: relationship with acute‐on‐chronic liver failure and prognosis. Gastroenterology. 2021;160(1):206‐218.e13. 32941879 10.1053/j.gastro.2020.08.054

[109] Acharya C , Bajaj JS . Altered microbiome in patients with cirrhosis and complications. Clin Gastroenterol Hepatol. 2019;17:307‐321. 30099098 10.1016/j.cgh.2018.08.008 PMC6314917

[110] Schneider KM , et al. Imbalanced gut microbiota fuels hepatocellular carcinoma development by shaping the hepatic inflammatory microenvironment. Nat Commun. 2022;13:3964. 35803930 10.1038/s41467-022-31312-5 PMC9270328

[111] Shen Y , Wu SD , Chen Y , et al. Alterations in gut microbiome and metabolomics in chronic hepatitis B infection‐associated liver disease and their impact on peripheral immune response. Gut Microbes. 2023;15:2155018. 36519342 10.1080/19490976.2022.2155018 PMC9757487

[112] Lu Y , Yuan X , Wang M , et al. Gut microbiota influence immunotherapy responses: mechanisms and therapeutic strategies. J Hematol Oncol. 2022;15:47. 35488243 10.1186/s13045-022-01273-9 PMC9052532

[113] McLane LM , Abdel‐Hakeem MS , Wherry EJ . CD8 T cell exhaustion during chronic viral infection and cancer. Annu Rev Immunol. 2019;37:457‐495. 30676822 10.1146/annurev-immunol-041015-055318

[114] Wang Q , Qin Y , Li B . CD8+ T cell exhaustion and cancer immunotherapy. Cancer Lett. 2023;559:216043. 36584935 10.1016/j.canlet.2022.216043

[115] Hashimoto M , Kamphorst AO , Im SJ , et al. CD8 T cell exhaustion in chronic infection and cancer: opportunities for interventions. Annu Rev Med. 2018;69:301‐318. 29414259 10.1146/annurev-med-012017-043208

[116] Jia L , Gao Y , He Y , Hooper JD , Yang P . HBV induced hepatocellular carcinoma and related potential immunotherapy. Pharmacol Res. 2020;159:104992. 32505833 10.1016/j.phrs.2020.104992

[117] Oura K , Morishita A , Tani J , Masaki T . Tumor immune microenvironment and immunosuppressive therapy in hepatocellular carcinoma: a review. Int J Mol Sci. 2021;22(11):5801. 34071550 10.3390/ijms22115801 PMC8198390

[118] Donne R , Lujambio A . The liver cancer immune microenvironment: therapeutic implications for hepatocellular carcinoma. Hepatology. 2023;77:1773‐1796. 35989535 10.1002/hep.32740 PMC9941399

[119] Llovet JM , Castet F , Heikenwalder M , et al. Immunotherapies for hepatocellular carcinoma. Nat Rev Clin Oncol. 2022;19:151‐172. 34764464 10.1038/s41571-021-00573-2

[120] Rizvi S , Wang J , El‐Khoueiry AB . Liver cancer immunity. *Hepatology*. 2020;73:86‐103. 10.1002/hep.31416 PMC8218340 32516437

[121] Yuen VW‐H , Wong CC‐L . Hypoxia‐inducible factors and innate immunity in liver cancer. J Clin Investig. 2020;130:5052‐5062. 32750043 10.1172/JCI137553 PMC7524494

[122] Pineiro Fernandez J , Luddy KA , Harmon C , O'Farrelly C . Hepatic tumor microenvironments and effects on NK cell phenotype and function. Int J Mol Sci. 2019;20(17):4131. 31450598 10.3390/ijms20174131 PMC6747260

[123] Saleh R , Elkord E . Acquired resistance to cancer immunotherapy: role of tumor‐mediated immunosuppression. Semin Cancer Biol. 2020;65:13‐27. 31362073 10.1016/j.semcancer.2019.07.017

[124] Chen J , Wang R , Liu Z , et al. Unbalanced glutamine partitioning between CD8T cells and cancer cells accompanied by immune cell dysfunction in hepatocellular carcinoma. Cells. 2022;11(23):3924. 36497182 10.3390/cells11233924 PMC9739589

[125] Lu Y , Yang A , Quan C , et al. A single‐cell atlas of the multicellular ecosystem of primary and metastatic hepatocellular carcinoma. Nat Commun. 2022;13:4594. 35933472 10.1038/s41467-022-32283-3 PMC9357016

[126] Pan B , Wang Z , Zhang X , et al. Targeted inhibition of RBPJ transcription complex alleviates the exhaustion of CD8(+) T cells in hepatocellular carcinoma. Commun Biol. 2023;6:123. 36717584 10.1038/s42003-023-04521-x PMC9887061

[127] Hung MH , Lee JS , Ma C , et al. Tumor methionine metabolism drives T‐cell exhaustion in hepatocellular carcinoma. Nat Commun. 2021;12:1455. 33674593 10.1038/s41467-021-21804-1 PMC7935900

[128] Wang X , He Q , Shen H , Lu XJ , Sun B . Genetic and phenotypic difference in CD8(+) T cell exhaustion between chronic hepatitis B infection and hepatocellular carcinoma. J Med Genet. 2019;56:18‐21. 29666149 10.1136/jmedgenet-2018-105267 PMC6327916

[129] Lequeux A , Noman MZ , Xiao M , et al. Impact of hypoxic tumor microenvironment and tumor cell plasticity on the expression of immune checkpoints. Cancer Lett. 2019;458:13‐20. 31136782 10.1016/j.canlet.2019.05.021

[130] Chen S , Gao Y , Wang Y , Daemen T . The combined signatures of hypoxia and cellular landscape provides a prognostic and therapeutic biomarker in hepatitis B virus‐related hepatocellular carcinoma. Int J Cancer. 2022;151:809‐824. 35467769 10.1002/ijc.34045 PMC9543189

[131] Mo Z , Liu D , Rong D , Zhang S . Hypoxic characteristic in the immunosuppressive microenvironment of hepatocellular carcinoma. Front Immunol. 2021;12:611058. 33679749 10.3389/fimmu.2021.611058 PMC7928397

[132] Wu Q , Zhou W , Yin S , et al. Blocking triggering receptor expressed on myeloid cells‐1‐positive tumor‐associated macrophages induced by hypoxia reverses immunosuppression and anti‐programmed cell death ligand 1 resistance in liver cancer. Hepatology. 2019;70:198‐214. 30810243 10.1002/hep.30593 PMC6618281

[133] Tran CW , Gold MJ , Garcia‐Batres C , et al. Hypoxia‐inducible factor 1 alpha limits dendritic cell stimulation of CD8 T cell immunity. PLoS One. 2020;15:e0244366. 33382742 10.1371/journal.pone.0244366 PMC7775062

[134] Dai X , Pi G , Yang SL , Chen GG , Liu LP , Dong HH . Association of PD‐L1 and HIF‐1α coexpression with poor prognosis in hepatocellular carcinoma. Transl Oncol. 2018;11:559‐566. 29525633 10.1016/j.tranon.2018.02.014 PMC5884219

[135] Gropper Y , Feferman T , Shalit T , et al. Culturing CTLs under hypoxic conditions enhances their cytolysis and improves their anti‐tumor function. Cell Rep. 2017;20:2547‐2555. 28903036 10.1016/j.celrep.2017.08.071

[136] Palazon A , Tyrakis PA , Macias D , et al. An HIF‐1alpha/VEGF‐A axis in cytotoxic t cells regulates tumor progression. Cancer Cell. 2017;32:669‐683. e665. 29136509 10.1016/j.ccell.2017.10.003 PMC5691891

[137] Starzer AM , Berghoff AS . New emerging targets in cancer immunotherapy: cD27 (TNFRSF7). ESMO Open. 2020;4:e000629. 32152062 10.1136/esmoopen-2019-000629 PMC7082637

[138] Veliça P , Cunha PP , Vojnovic N , et al. Modified hypoxia‐inducible factor expression in CD8+ T cells increases antitumor efficacy. Cancer Immunol Res. 2021;9:401‐414. 33602720 10.1158/2326-6066.CIR-20-0561 PMC7611205

[139] Bao MH‐R , Wong CC‐L . Hypoxia, metabolic reprogramming, and drug resistance in liver cancer. Cells. 2021;10(7):1715. 34359884 10.3390/cells10071715 PMC8304710

[140] Zhu H , Wang DD , Yuan T , et al. Multikinase inhibitor CT‐707 targets liver cancer by interrupting the hypoxia‐activated IGF‐1R‐YAP axis. Cancer Res. 2018;78:3995‐4006. 29669759 10.1158/0008-5472.CAN-17-1548

[141] Warburg O , Wind F , Negelein E . The metabolism of tumors in the body. J Gen Physiol. 1927;8:519‐530. 19872213 10.1085/jgp.8.6.519 PMC2140820

[142] Bartman CR , Weilandt DR , Shen Y , et al. Slow TCA flux and ATP production in primary solid tumours but not metastases. Nature. 2023;614:349‐357. 36725930 10.1038/s41586-022-05661-6 PMC10288502

[143] Xu Y , Hao X , Ren Y , et al. Research progress of abnormal lactate metabolism and lactate modification in immunotherapy of hepatocellular carcinoma. Front Oncol. 2023;12:1063423. 36686771 10.3389/fonc.2022.1063423 PMC9853001

[144] Zhang Y , Zhai Z , Duan J , et al. Lactate: the mediator of metabolism and immunosuppression. Front Endocrinol. 2022;13:901495. 10.3389/fendo.2022.901495 PMC9218951 35757394

[145] Li Y , Mo H , Wu S , Liu X , Tu K . A novel lactate metabolism‐related gene signature for predicting clinical outcome and tumor microenvironment in hepatocellular carcinoma. Front Cell Dev Biol. 2022;9:801959. 35047511 10.3389/fcell.2021.801959 PMC8762248

[146] Nakagawa Y , Negishi Y , Shimizu M , et al. Effects of extracellular pH and hypoxia on the function and development of antigen‐specific cytotoxic T lymphocytes. Immunol Lett. 2015;167:72‐86. 26209187 10.1016/j.imlet.2015.07.003

[147] Elia I , Rowe JH , Johnson S , et al. Tumor cells dictate anti‐tumor immune responses by altering pyruvate utilization and succinate signaling in CD8+ T cells. Cell Metab. 2022;34(8):1137‐1150.e6. 35820416 10.1016/j.cmet.2022.06.008 PMC9357162

[148] Song BS , Moon JS , Tian J , et al. Mitoribosomal defects aggravate liver cancer via aberrant glycolytic flux and T cell exhaustion. J Immunother Cancer. 2022;10(5):e004337. 35580931 10.1136/jitc-2021-004337 PMC9114962

[149] Kumagai S , Koyama S , Itahashi K , et al. Lactic acid promotes PD‐1 expression in regulatory T cells in highly glycolytic tumor microenvironments. Cancer Cell. 2022;40(2):201‐218.e9. 35090594 10.1016/j.ccell.2022.01.001

[150] Johnson S , Haigis MC , Dougan SK . Dangerous dynamic duo: lactic acid and PD‐1 blockade. Cancer Cell. 2022;40:127‐130. 35093211 10.1016/j.ccell.2022.01.008

[151] Wang J , Wang H , Gao M , et al. The regulation of amino acid metabolism in tumor cell death: from the perspective of physiological functions. Apoptosis. 2023;28:1304‐1314. 37523039 10.1007/s10495-023-01875-9

[152] Zhao Y , Zhang J , Wang S , Jiang Q , Xu K . Identification and validation of a nine‐gene amino acid metabolism‐related risk signature in HCC. Front Cell Dev Biol. 2021;9:731790. 34557495 10.3389/fcell.2021.731790 PMC8452960

[153] Li Y , Wu Y , Hu Y . Metabolites in the tumor microenvironment reprogram functions of immune effector cells through epigenetic modifications. Front Immunol. 2021;12:641883. 33927716 10.3389/fimmu.2021.641883 PMC8078775

[154] Dai W , Xu L , Yu X , et al. OGDHL silencing promotes hepatocellular carcinoma by reprogramming glutamine metabolism. J Hepatol. 2020;72:909‐923. 31899205 10.1016/j.jhep.2019.12.015

[155] Wang Y , Bai C , Ruan Y , et al. Coordinative metabolism of glutamine carbon and nitrogen in proliferating cancer cells under hypoxia. Nat Commun. 2019;10:201. 30643150 10.1038/s41467-018-08033-9 PMC6331631

[156] Li Y , Li B , Xu Y , et al. GOT2 silencing promotes reprogramming of glutamine metabolism and sensitizes hepatocellular carcinoma to glutaminase inhibitors. Cancer Res. 2022;82:3223‐3235. 35895805 10.1158/0008-5472.CAN-22-0042

[157] Leone RD , Zhao L , Englert JM , et al. Glutamine blockade induces divergent metabolic programs to overcome tumor immune evasion. Science. 2019;366:1013‐1021. 31699883 10.1126/science.aav2588 PMC7023461

[158] Wang W , Guo MN , Li N , Pang DQ , Wu JH . Glutamine deprivation impairs function of infiltrating CD8(+) T cells in hepatocellular carcinoma by inducing mitochondrial damage and apoptosis. World J Gastrointest Oncol. 2022;14:1124‐1140. 35949216 10.4251/wjgo.v14.i6.1124 PMC9244988

[159] He W , Hu Y , Chen D , et al. Hepatocellular carcinoma‐infiltrating γδ T cells are functionally defected and allogenic Vδ2+ γδ T cell can be a promising complement. Clin Transl Med. 2022;12:e800. 35390227 10.1002/ctm2.800 PMC8989380

[160] Krishnamurthy S , Gilot D , Ahn SB , et al. Involvement of kynurenine pathway in hepatocellular carcinoma. Cancers (Basel). 2021;13:(20):5180. 34680327 10.3390/cancers13205180 PMC8533819

[161] Kim M , Tomek PT . A rheostat of cancer immune escape mediated by immunosuppressive enzymes IDO1 and TDO. Front Immunol. 2021;12:636081. 33708223 10.3389/fimmu.2021.636081 PMC7940516

[162] Hoffmann D , Dvorakova T , Stroobant V , et al. Tryptophan 2,3‐dioxygenase expression identified in human hepatocellular carcinoma cells and in intratumoral pericytes of most cancers. Cancer Immunol Res. 2020;8:19‐31. 31806639 10.1158/2326-6066.CIR-19-0040

[163] Yu L , Lu J , Du W . Tryptophan metabolism in digestive system tumors: unraveling the pathways and implications. Cell Commun Signal. 2024;22:174. 38462620 10.1186/s12964-024-01552-7 PMC10926624

[164] Liang X , Gao H , Xiao J , et al. Abrine, an IDO1 inhibitor, suppresses the immune escape and enhances the immunotherapy of anti‐PD‐1 antibody in hepatocellular carcinoma. Front Immunol. 2023;14:1185985. 37334368 10.3389/fimmu.2023.1185985 PMC10272936

[165] Tang K , Wu Y‐H , Song Y , Yu B . Indoleamine 2,3‐dioxygenase 1 (IDO1) inhibitors in clinical trials for cancer immunotherapy. J Hematol Oncol. 2021;14(1):68. 33883013 10.1186/s13045-021-01080-8 PMC8061021

[166] Li S , Han X , Lyu N , et al. Mechanism and prognostic value of indoleamine 2,3‐dioxygenase 1 expressed in hepatocellular carcinoma. Cancer Sci. 2018;109:3726‐3736. 30264546 10.1111/cas.13811 PMC6272112

[167] Zhang R , Li T , Wang W , et al. Indoleamine 2, 3‐dioxygenase 1 and CD8 expression profiling revealed an immunological subtype of colon cancer with a poor prognosis. Front Oncol. 2020;10:594098. 33425745 10.3389/fonc.2020.594098 PMC7793995

[168] Zhou Q‐H , Han H , Lu JB , et al. Up‐regulation of indoleamine 2,3‐dioxygenase 1 (IDO1) expression and catalytic activity is associated with immunosuppression and poor prognosis in penile squamous cell carcinoma patients. Cancer Commun (Lond). 2020;40(1):3‐15. 32125093 10.1002/cac2.12001 PMC7163927

[169] Li S , Li L , Wu J , et al. TDO promotes hepatocellular carcinoma progression. Onco Targets Ther. 2020;13:5845‐5855. 32606795 10.2147/OTT.S252929 PMC7311207

[170] Greene LI , Bruno TC , Christenson JL , et al. A role for tryptophan‐2,3‐dioxygenase in CD8 T‐cell suppression and evidence of tryptophan catabolism in breast cancer patient plasma. Mol Cancer Res. 2019;17:131‐139. 30143553 10.1158/1541-7786.MCR-18-0362 PMC6318037

[171] Marszalek‐Grabska M , Walczak K , Gawel K , et al. Kynurenine emerges from the shadows—current knowledge on its fate and function. Pharmacol Ther. 2021;225:107845. 33831481 10.1016/j.pharmthera.2021.107845

[172] Wang Z , Zhang Y , Liao Z , Huang M , Shui X . The potential of aryl hydrocarbon receptor as receptors for metabolic changes in tumors. Front Oncol. 2024;14:1328606. 38434684 10.3389/fonc.2024.1328606 PMC10904539

[173] Zhu Q , Ma Y , Liang J , et al. AHR mediates the aflatoxin B1 toxicity associated with hepatocellular carcinoma. Signal Transduct Target Ther. 2021;6(1):299. 34373448 10.1038/s41392-021-00713-1 PMC8352983

[174] Celik‐Turgut G , Olmez N , Koc T , et al. Role of AHR, NF‐kB and CYP1A1 crosstalk with the X protein of Hepatitis B virus in hepatocellular carcinoma cells. Gene. 2023;853:147099. 36476661 10.1016/j.gene.2022.147099

[175] Chen C‐T , Wu PH , Hu CC , et al. Aberrant upregulation of indoleamine 2,3‐dioxygenase 1 promotes proliferation and metastasis of hepatocellular carcinoma cells via coordinated activation of AhR and β‐Catenin signaling. Int J Mol Sci. 2021;22(21):11661. 34769098 10.3390/ijms222111661 PMC8583706

[176] Liu Y , Zhou N , Zhou L , et al. IL‐2 regulates tumor‐reactive CD8+ T cell exhaustion by activating the aryl hydrocarbon receptor. Nat Immunol. 2021;22:358‐369. 33432230 10.1038/s41590-020-00850-9

[177] Shi J , Chen C , Ju R , et al. Carboxyamidotriazole combined with IDO1‐Kyn‐AhR pathway inhibitors profoundly enhances cancer immunotherapy. J Immunother Cancer. 2019;7. 10.1186/s40425-019-0725-7 PMC6740021 31511064

[178] Tripodi F , Badone B , Vescovi M , et al. Methionine supplementation affects metabolism and reduces tumor aggressiveness in liver cancer cells. Cells. 2020;9(1):246. 33207837 10.3390/cells9112491 PMC7696226

[179] Wanders D , Hobson K , Ji X . Methionine restriction and cancer biology. Nutrients. 2020;12(3):684. 32138282 10.3390/nu12030684 PMC7146589

[180] Cavuoto P , Fenech MF . A review of methionine dependency and the role of methionine restriction in cancer growth control and life‐span extension. Cancer Treat Rev. 2012;38:726‐736. 22342103 10.1016/j.ctrv.2012.01.004

[181] Nishizaki T , Matsumata T , Taketomi A , Yamamoto K , Sugimachi K . Levels of amino acids in human hepatocellular carcinoma and adjacent liver tissue. Nutr Cancer. 1995;23:85‐90. 7739918 10.1080/01635589509514364

[182] Sanderson SM , Gao X , Dai Z , Locasale JW . Methionine metabolism in health and cancer: a nexus of diet and precision medicine. Nat Rev Cancer. 2019;19:625‐637. 31515518 10.1038/s41568-019-0187-8

[183] Pascale RM , Simile MM , Calvisi DF , Feo CF , Feo F . S‐adenosylmethionine: from the discovery of its inhibition of tumorigenesis to its use as a therapeutic agent. Cells. 2022;11(3):409. 35159219 10.3390/cells11030409 PMC8834208

[184] Li F , Liu P , Mi W , et al. Blocking methionine catabolism induces senescence and confers vulnerability to GSK3 inhibition in liver cancer. Nat Cancer. 2024;5:131‐146. 38168934 10.1038/s43018-023-00671-3 PMC11277537

[185] Bian Y , Li W , Kremer DM , et al. Cancer SLC43A2 alters T cell methionine metabolism and histone methylation. Nature. 2020;585:277‐282. 32879489 10.1038/s41586-020-2682-1 PMC7486248

[186] Liao Y , Weng J , Chen L , et al. Comprehensive analysis of SLC43A2 on the tumor immune microenvironment and prognosis of liver hepatocellular carcinoma. Front Genet. 2022;13:911378. 36186480 10.3389/fgene.2022.911378 PMC9523210

[187] Gao X , Sanderson SM , Dai Z , et al. Dietary methionine influences therapy in mouse cancer models and alters human metabolism. Nature. 2019;572:397‐401. 31367041 10.1038/s41586-019-1437-3 PMC6951023

[188] Li T , Tan YT , Chen YX , et al. Methionine deficiency facilitates antitumour immunity by altering m(6)A methylation of immune checkpoint transcripts. Gut. 2023;72:501‐511. 35803704 10.1136/gutjnl-2022-326928 PMC9933173

[189] Xia L , Oyang L , Lin J , et al. The cancer metabolic reprogramming and immune response. Mol Cancer. 2021;20:28. 33546704 10.1186/s12943-021-01316-8 PMC7863491

[190] Mossmann D , Müller C , Park S , et al. Arginine reprograms metabolism in liver cancer via RBM39. Cell. 2023;186:5068‐5083. e5023. 37804830 10.1016/j.cell.2023.09.011 PMC10642370

[191] Missiaen R , Anderson NM , Kim LC , et al. GCN2 inhibition sensitizes arginine‐deprived hepatocellular carcinoma cells to senolytic treatment. Cell Metab. 2022;34:1151‐1167. e1157. 35839757 10.1016/j.cmet.2022.06.010 PMC9357184

[192] Glöckner HJ , Martinenaite E , Landkildehus Lisle T , et al. Arginase‐1 specific CD8+ T cells react toward malignant and regulatory myeloid cells. OncoImmunology. 2024;13(1):2318053. 38404966 10.1080/2162402X.2024.2318053 PMC10885169

[193] Heim K , Neumann‐Haefelin C , Thimme R , Hofmann M . Heterogeneity of HBV‐specific CD8(+) T‐cell failure: implications for immunotherapy. Front Immunol. 2019;10:2240. 31620140 10.3389/fimmu.2019.02240 PMC6763562

[194] Chang CH , Qiu J , O'Sullivan D , et al. Metabolic competition in the tumor microenvironment is a driver of cancer progression. Cell. 2015;162:1229‐1241. 26321679 10.1016/j.cell.2015.08.016 PMC4864363

[195] Tang Q , Chen Y , Li X , et al. The role of PD‐1/PD‐L1 and application of immune‐checkpoint inhibitors in human cancers. Front Immunol. 2022;13:964442. 36177034 10.3389/fimmu.2022.964442 PMC9513184

[196] Pardoll DM . The blockade of immune checkpoints in cancer immunotherapy. Nat Rev Cancer. 2012;12:252‐264. 22437870 10.1038/nrc3239 PMC4856023

[197] Inada Y , Mizukoshi E , Seike T , et al. Characteristics of immune response to tumor‐associated antigens and immune cell profile in patients with hepatocellular carcinoma. Hepatology. 2019;69:653‐665. 30102778 10.1002/hep.30212

[198] Kim H‐D , Song GW , Park S , et al. Association between expression level of PD1 by tumor‐infiltrating CD8+ T cells and features of hepatocellular carcinoma. Gastroenterology. 2018;155(6):1936‐1950.e17. 30145359 10.1053/j.gastro.2018.08.030

[199] Ma J , Zheng B , Goswami S , et al. PD1Hi CD8+ T cells correlate with exhausted signature and poor clinical outcome in hepatocellular carcinoma. J Immunother Cancer. 2019;7:331. 31783783 10.1186/s40425-019-0814-7 PMC6884778

[200] Wang X , He Q , Shen H , et al. TOX promotes the exhaustion of antitumor CD8(+) T cells by preventing PD1 degradation in hepatocellular carcinoma. J Hepatol. 2019;71:731‐741. 31173813 10.1016/j.jhep.2019.05.015

[201] Walunas TL , et al. CTLA‐4 can function as a negative regulator of T cell activation. Immunity. 1994;1:405‐413. 21934098

[202] Krummel MF , Allison JP . CD28 and CTLA‐4 have opposing effects on the response of T cells to stimulation. J Exp Med. 1995;182:459‐465. 7543139 10.1084/jem.182.2.459 PMC2192127

[203] Zhao Z , Wang C , Chu P , Lu X . Key genes associated with tumor‐infiltrating non‐regulatory CD4‐ and CD8‐Positive T cells in microenvironment of hepatocellular carcinoma. Biochem Genet. 2022;60:1762‐1780. 35092558 10.1007/s10528-021-10175-3 PMC9470630

[204] Wang L , Li N , Fan X , et al. Circulating CTLA‐4 levels and CTLA4 polymorphisms associate with disease condition and progression and hepatocellular carcinoma patients' survival in chronic hepatitis B virus infection. Int Immunopharmacol. 2020;82:106377. 32163858 10.1016/j.intimp.2020.106377

[205] Wang Z , Wang Y , Gao P , Ding J . Immune checkpoint inhibitor resistance in hepatocellular carcinoma. Cancer Lett. 2023;555:216038. 36529238 10.1016/j.canlet.2022.216038

[206] Kang Y , Cai Y , Yang Y . The gut microbiome and hepatocellular carcinoma: implications for early diagnostic biomarkers and novel therapies. Liver Cancer. 2022;11:113‐125. 35634424 10.1159/000521358 PMC9109080

[207] Yu L‐X , Schwabe RF . The gut microbiome and liver cancer: mechanisms and clinical translation. Nat Rev Gastroenterol Hepatol. 2017;14:527‐539. 28676707 10.1038/nrgastro.2017.72 PMC6467288

[208] Liu Q , Li F , Zhuang Y , et al. Alteration in gut microbiota associated with hepatitis B and non‐hepatitis virus related hepatocellular carcinoma. Gut Pathog. 2019;11:1. 30675188 10.1186/s13099-018-0281-6 PMC6337822

[209] Ren Z , Li A , Jiang J , et al. Gut microbiome analysis as a tool towards targeted non‐invasive biomarkers for early hepatocellular carcinoma. Gut. 2019;68:1014‐1023. 30045880 10.1136/gutjnl-2017-315084 PMC6580753

[210] Huang H , Ren Z , Gao X , et al. Integrated analysis of microbiome and host transcriptome reveals correlations between gut microbiota and clinical outcomes in HBV‐related hepatocellular carcinoma. Genome Med. 2020;12:102. 33225985 10.1186/s13073-020-00796-5 PMC7682083

[211] Rajapakse J , Khatiwada S , Akon AC , et al. Unveiling the complex relationship between gut microbiota and liver cancer: opportunities for novel therapeutic interventions. Gut Microbes. 2023;15:2240031. 37615334 10.1080/19490976.2023.2240031 PMC10454000

[212] Zeng Y , Chen S , Fu Y , et al. Gut microbiota dysbiosis in patients with hepatitis B virus‐induced chronic liver disease covering chronic hepatitis, liver cirrhosis and hepatocellular carcinoma. J Viral Hepat. 2020;27:143‐155. 31600845 10.1111/jvh.13216

[213] Ma H , Yang L , Liang Y , et al. B. thetaiotaomicron‐derived acetic acid modulate immune microenvironment and tumor growth in hepatocellular carcinoma. Gut Microbes. 2024;16:2297846. 38270111 10.1080/19490976.2023.2297846 PMC10813637

[214] Gopalakrishnan V , Spencer CN , Nezi L , et al. Gut microbiome modulates response to anti‐PD‐1 immunotherapy in melanoma patients. Science. 2018;359:97‐103. 29097493 10.1126/science.aan4236 PMC5827966

[215] Zheng Y , Wang T , Tu X , et al. Gut microbiome affects the response to anti‐PD‐1 immunotherapy in patients with hepatocellular carcinoma. J Immunother Cancer. 2019;7:193. 31337439 10.1186/s40425-019-0650-9 PMC6651993

[216] Mao J , Wang D , Long J , et al. Gut microbiome is associated with the clinical response to anti‐PD‐1 based immunotherapy in hepatobiliary cancers. J Immunother Cancer. 2021;9(12):e003334. 34873013 10.1136/jitc-2021-003334 PMC8650503

[217] Pan B , Chen Z , Zhang X , et al. 2,5‐Dimethylcelecoxib alleviated NK and T‐cell exhaustion in hepatocellular carcinoma via the gastrointestinal microbiota‐AMPK‐mTOR axis. J ImmunotherCancer. 2023;11(6):e006817. 10.1136/jitc-2023-006817 PMC10277542 37316264

[218] He Y , Fu L , Li Y , et al. Gut microbial metabolites facilitate anticancer therapy efficacy by modulating cytotoxic CD8+ T cell immunity. Cell Metab. 2021;33:988‐1000. e1007. 33761313 10.1016/j.cmet.2021.03.002

[219] Sangro B , Sarobe P , Hervas‐Stubbs S , Melero I . Advances in immunotherapy for hepatocellular carcinoma. Nat Rev Gastroenterol Hepatol. 2021;18:525‐543. 33850328 10.1038/s41575-021-00438-0 PMC8042636

[220] Liu JKH , Irvine AF , Jones RL , Samson A . Immunotherapies for hepatocellular carcinoma. Cancer Med. 2022;11:571‐591. 34953051 10.1002/cam4.4468 PMC8817091

[221] El‐Khoueiry AB , Sangro B , Yau T , et al. Nivolumab in patients with advanced hepatocellular carcinoma (CheckMate 040): an open‐label, non‐comparative, phase 1/2 dose escalation and expansion trial. Lancet. 2017;389:2492‐2502. 28434648 10.1016/S0140-6736(17)31046-2 PMC7539326

[222] Chen Y , Hu H , Yuan X , Fan X , Zhang C , et al. Advances in immune checkpoint inhibitors for advanced hepatocellular carcinoma. Front Immunol. 2022;13:896752. 35757756 10.3389/fimmu.2022.896752 PMC9226303

[223] Agdashian D , ElGindi M , Xie C , et al. The effect of anti‐CTLA4 treatment on peripheral and intra‐tumoral T cells in patients with hepatocellular carcinoma. Cancer Immunol Immunother. 2019;68:599‐608. 30688989 10.1007/s00262-019-02299-8 PMC6662600

[224] Rimassa L , Finn RS , Sangro B . Combination immunotherapy for hepatocellular carcinoma. J Hepatol. 2023;79:506‐515. 36933770 10.1016/j.jhep.2023.03.003

[225] Kelley RK , Sangro B , Harris W , et al. Safety, efficacy, and pharmacodynamics of tremelimumab plus durvalumab for patients with unresectable hepatocellular carcinoma: randomized expansion of a phase I/II study. J Clin Oncol. 2021;39:2991‐3001. 34292792 10.1200/JCO.20.03555 PMC8445563

[226] Ren Z , Xu J , Bai Y , et al. Sintilimab plus a bevacizumab biosimilar (IBI305) versus sorafenib in unresectable hepatocellular carcinoma (ORIENT‐32): a randomised, open‐label, phase 2–3 study. Lancet Oncol. 2021;22:977‐990. 34143971 10.1016/S1470-2045(21)00252-7

[227] Hu B , Yu M , Ma X , et al. IFNα potentiates anti‐PD‐1 efficacy by remodeling glucose metabolism in the hepatocellular carcinoma microenvironment. Cancer Discov. 2022;12:1718‐1741. 35412588 10.1158/2159-8290.CD-21-1022

[228] Mizukoshi E , Kaneko S . Immune cell therapy for hepatocellular carcinoma. J Hematol Oncol. 2019;12:52. 10.1186/s13045-019-0742-5 PMC6542133 31142330

[229] Jiang X , Xu J , Liu M , et al. Adoptive CD8+ T cell therapy against cancer:challenges and opportunities. Cancer Lett. 2019;462:23‐32. 31356845 10.1016/j.canlet.2019.07.017

[230] Liu Q , Tian Y , Li Y , et al. In vivo therapeutic effects of affinity‐improved‐TCR engineered T‐cells on HBV‐related hepatocellular carcinoma. J Immunother Cancer. 2020;8(2):e001748. 33323464 10.1136/jitc-2020-001748 PMC7745518

[231] Meng F , Zhao J , Tan AT , et al. Immunotherapy of HBV‐related advanced hepatocellular carcinoma with short‐term HBV‐specific TCR expressed T cells: results of dose escalation, phase I trial. Hepatol Int. 2021;15:1402‐1412. 34850325 10.1007/s12072-021-10250-2 PMC8651587

[232] Liu Z , Liu X , Liang J , et al. Immunotherapy for hepatocellular carcinoma: current status and future prospects. Front Immunol. 2021;12:765101. 34675942 10.3389/fimmu.2021.765101 PMC8524467

[233] Sterner RC , Sterner RM . CAR‐T cell therapy: current limitations and potential strategies. Blood Cancer J. 2021;11(4):69. 33824268 10.1038/s41408-021-00459-7 PMC8024391

[234] Golubovskaya V , Wu L . Different subsets of T cells, memory, effector functions, and CAR‐T immunotherapy. Cancers. 2016;8(3):36. 26999211 10.3390/cancers8030036 PMC4810120

[235] Patel K , Lamm R , Altshuler P , Dang H , Shah AP . Hepatocellular carcinoma‐the influence of immunoanatomy and the role of immunotherapy. Int J Mol Sci. 2020;21(18):6757. 32942580 10.3390/ijms21186757 PMC7555667

[236] Chen Y , E CY , Gong ZW , et al. Chimeric antigen receptor‐engineered T‐cell therapy for liver cancer. Hepatobiliary Pancreat Dis Int. 2018;17:301‐309. 29861325 10.1016/j.hbpd.2018.05.005

[237] Zheng X , Liu X , Lei Y , Wang G , Liu M . Glypican‐3: a novel and promising target for the treatment of hepatocellular carcinoma. Front Oncol. 2022;12:824208. 35251989 10.3389/fonc.2022.824208 PMC8889910

[238] Li D , Li N , Zhang YF , et al. Persistent polyfunctional chimeric antigen receptor T cells that target glypican 3 eliminate orthotopic hepatocellular carcinomas in mice. Gastroenterology. 2020;158:2250‐2265. e2220. 32060001 10.1053/j.gastro.2020.02.011 PMC7282931

[239] Zhai B , Shi D , Gao H , et al. A phase I study of anti‐GPC3 chimeric antigen receptor modified T cells (GPC3 CAR‐T) in Chinese patients with refractory or relapsed GPC3+ hepatocellular carcinoma (r/r GPC3+ HCC). J Clin Oncol. 2017;35:3049‐3049.

[240] Sun B , Yang D , Dai H , et al. Eradication of hepatocellular carcinoma by NKG2D‐Based CAR‐T Cells. Cancer Immunol Res. 2019;7:1813‐1823. 31484657 10.1158/2326-6066.CIR-19-0026

[241] Wang X , Wu Z , Qiu W , Chen P , Xu X , Han W . Programming CAR T cells to enhance anti‐tumor efficacy through remodeling of the immune system. Front Med. 2020;14:726‐745. 32794014 10.1007/s11684-020-0746-0

[242] Hu Z , Ott PA , Wu CJ . Towards personalized, tumour‐specific, therapeutic vaccines for cancer. Nat Rev Immunol. 2018;18:168‐182. 29226910 10.1038/nri.2017.131 PMC6508552

[243] Saxena M , van der Burg SH , Melief CJM , Bhardwaj N . Therapeutic cancer vaccines. Nat Rev Cancer. 2021;21:360‐378. 33907315 10.1038/s41568-021-00346-0

[244] Melief CJM , et al. Therapeutic cancer vaccines. J Clin Invest. 2015;125:3401‐3412. 26214521 10.1172/JCI80009 PMC4588240

[245] Hu X , Chen R , Wei Q , Xu X . The landscape of alpha fetoprotein in hepatocellular carcinoma: where are we? Int J Biol Sci. 2022;18:536‐551. 35002508 10.7150/ijbs.64537 PMC8741863

[246] Taniguchi M , Mizuno S , Yoshikawa T , et al. Peptide vaccine as an adjuvant therapy for glypican‐3‐positive hepatocellular carcinoma induces peptide‐specific CTLs and improves long prognosis. Cancer Sci. 2020;111:2747‐2759. 32449239 10.1111/cas.14497 PMC7419030

[247] Lu X , Deng S , Xu J , et al. Combination of AFP vaccine and immune checkpoint inhibitors slows hepatocellular carcinoma progression in preclinical models. J Clin Invest. 2023;133(11):e163291. 37040183 10.1172/JCI163291 PMC10231990

[248] Chen H , Li Z , Qiu L , et al. Personalized neoantigen vaccine combined with PD‐1 blockade increases CD8+ tissue‐resident memory T‐cell infiltration in preclinical hepatocellular carcinoma models. J Immunother Cancer. 2022;10(9):e004389. 36113894 10.1136/jitc-2021-004389 PMC9486396

[249] Caraballo Galva LD , Cai L , Shao Y , He Y . Engineering T cells for immunotherapy of primary human hepatocellular carcinoma. J Genet Genomics. 2020;47:1‐15. 32089500 10.1016/j.jgg.2020.01.002 PMC7093251

[250] Luo W , Guo S , Zhou Y , et al. Hepatocellular carcinoma: how the gut microbiota contributes to pathogenesis, diagnosis, and therapy. Front Microbiol. 2022;13:873160. 35572649 10.3389/fmicb.2022.873160 PMC9092458

[251] Yoshimoto S , Loo TM , Atarashi K , et al. Obesity‐induced gut microbial metabolite promotes liver cancer through senescence secretome. Nature. 2013;499:97‐101. 23803760 10.1038/nature12347

[252] Cheung KS , Lam LK , Seto WK , Leung WK . Use of antibiotics during immune checkpoint inhibitor treatment is associated with lower survival in hepatocellular carcinoma. Liver Cancer. 2021;10:606‐614. 34950183 10.1159/000518090 PMC8647068

[253] Zhou A , Tang L , Zeng S , Lei Y , Yang S , Tang B . Gut microbiota: a new piece in understanding hepatocarcinogenesis. Cancer Lett. 2020;474:15‐22. 31917160 10.1016/j.canlet.2020.01.002

[254] Zhang HL , Yu LX , Yang W , et al. Profound impact of gut homeostasis on chemically‐induced pro‐tumorigenic inflammation and hepatocarcinogenesis in rats. J Hepatol. 2012;57:803‐812. 22727732 10.1016/j.jhep.2012.06.011

[255] Zhang Q , Huang H , Zheng F , et al. Resveratrol exerts antitumor effects by downregulating CD8+CD122+ Tregs in murine hepatocellular carcinoma. Oncoimmunology. 2020;9:1829346. 33150044 10.1080/2162402X.2020.1829346 PMC7588216

[256] Fernandes MR , Aggarwal P , Costa RGF , Cole AM , Trinchieri G . Targeting the gut microbiota for cancer therapy. Nat Rev Cancer. 2022;22:703‐722. 36253536 10.1038/s41568-022-00513-x

[257] Cammarota G , Ianiro G , Tilg H , et al. European consensus conference on faecal microbiota transplantation in clinical practice. Gut. 2017;66:569‐580. 28087657 10.1136/gutjnl-2016-313017 PMC5529972

[258] Osman M , Budree S , Kelly CR , et al. Effectiveness and safety of fecal microbiota transplantation for clostridioides difficile infection: results from a 5344‐patient cohort study. Gastroenterology. 2022;163:319‐322. 35398345 10.1053/j.gastro.2022.03.051

[259] Zhao Y , Gong C , Xu J , et al. Research progress of fecal microbiota transplantation in liver diseases. J Clin Med. 2023;12(4):1683. 36836218 10.3390/jcm12041683 PMC9960958

[260] Davar D , Dzutsev AK , McCulloch JA , et al. Fecal microbiota transplant overcomes resistance to anti‐PD‐1 therapy in melanoma patients. Science (New York, NY). 2021;371:595‐602. 10.1126/science.abf3363 PMC8097968 33542131

[261] Porcari S , Benech N , Valles‐Colomer M , et al. Key determinants of success in fecal microbiota transplantation: from microbiome to clinic. Cell Host Microbe. 2023;31:712‐733. 37167953 10.1016/j.chom.2023.03.020

[262] Marcella C , Cui B , Kelly CR , et al. Systematic review: the global incidence of faecal microbiota transplantation‐related adverse events from 2000 to 2020. Aliment Pharmacol Ther. 2021;53:33‐42. 33159374 10.1111/apt.16148

[263] Liu X , Liu M , Zhao M , et al. Fecal microbiota transplantation for the management of autoimmune diseases: potential mechanisms and challenges. J Autoimmun. 2023;141:103109. 37690971 10.1016/j.jaut.2023.103109

[264] Lee MS , Ryoo BY , Hsu CH , et al. Atezolizumab with or without bevacizumab in unresectable hepatocellular carcinoma (GO30140): an open‐label, multicentre, phase 1b study. Lancet Oncol. 2020;21:808‐820. 32502443 10.1016/S1470-2045(20)30156-X

[265] Zhu AX , Finn RS , Edeline J , et al. Pembrolizumab in patients with advanced hepatocellular carcinoma previously treated with sorafenib (KEYNOTE‐224): a non‐randomised, open‐label phase 2 trial. Lancet Oncol. 2018;19:940‐952. 29875066 10.1016/S1470-2045(18)30351-6

[266] Sangro B , Gomez‐Martin C , de la Mata M , et al. A clinical trial of CTLA‐4 blockade with tremelimumab in patients with hepatocellular carcinoma and chronic hepatitis C. J Hepatol. 2013;59:81‐88. 23466307 10.1016/j.jhep.2013.02.022

[267] Li Z , Zhang Y , Hong W , et al. Gut microbiota modulate radiotherapy‐associated antitumor immune responses against hepatocellular carcinoma Via STING signaling. Gut Microbes. 2022;14:2119055. 36093568 10.1080/19490976.2022.2119055 PMC9467592

[268] Liang M , Liwen Z , Jianguo S , et al. Fecal microbiota transplantation controls progression of experimental autoimmune hepatitis in mice by modulating the TFR/TFH immune imbalance and intestinal microbiota composition. Front Immunol. 2021;12:728723. 34912328 10.3389/fimmu.2021.728723 PMC8667314

[269] Xue R , Zhang Q , Cao Q , et al. Liver tumour immune microenvironment subtypes and neutrophil heterogeneity. Nature. 2022;612:141‐147. 36352227 10.1038/s41586-022-05400-x

[270] Sun Y , Wu L , Zhong Y , et al. Single‐cell landscape of the ecosystem in early‐relapse hepatocellular carcinoma. Cell. 2021;184:404‐421. e416. 33357445 10.1016/j.cell.2020.11.041

[271] Lim CJ , Lee YH , Pan L , et al. Multidimensional analyses reveal distinct immune microenvironment in hepatitis B virus‐related hepatocellular carcinoma. Gut. 2019;68:916‐927. 29970455 10.1136/gutjnl-2018-316510

[272] Ma L , Hernandez MO , Zhao Y , et al. Tumor cell biodiversity drives microenvironmental reprogramming in liver cancer. Cancer Cell. 2019;36:418‐430. e416. 31588021 10.1016/j.ccell.2019.08.007 PMC6801104

[273] Sepich‐Poore GD , Zitvogel L , Straussman R , et al. The microbiome and human cancer. Science. 2021;371(6536):eabc4552.33766858

[274] De Crignis E , Hossain T , Romal S , et al. Application of human liver organoids as a patient‐derived primary model for HBV infection and related hepatocellular carcinoma. Elife. 2021;10:e60747. 34328417 10.7554/eLife.60747 PMC8384419

[275] Chen L , Wei X , Gu D , Xu Y , Zhou H . Human liver cancer organoids: biological applications, current challenges, and prospects in hepatoma therapy. Cancer Lett. 2023;555:216048. 36603689 10.1016/j.canlet.2022.216048

[276] Prior N , Inacio P , Huch M . Liver organoids: from basic research to therapeutic applications. Gut. 2019;68:2228‐2237. 31300517 10.1136/gutjnl-2019-319256 PMC6872443

